# Supplementary material for: Algorithmic discovery of dynamic models from infectious disease data
Source: Sci Rep. 2020 Apr 27;10:7061. doi: 10.1038/s41598-020-63877-w (PMC7184751; doi:10.1038/s41598-020-63877-w)
Supplement: Supplementary file 1 — Supplementary Appendix. [file 41598_2020_63877_MOESM1_ESM.pdf]

Supplementary Appendix: Algorithmic Discovery of Dynamic Models from Infectious Disease Data

Jonathan Horrocks[1], Chris T. Bauch[1,*]
[1]Department of Applied Mathematics, University of Waterloo
*correspondence: cbauch@uwaterloo.ca

1 Model rediscovery from simulated data

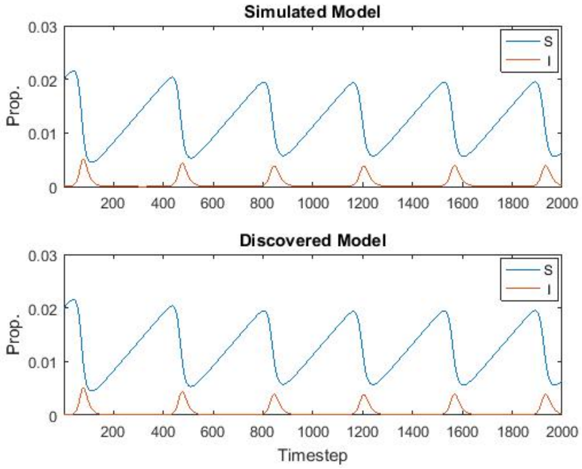

(a) Time-series

''	'Sdot'	'Idot'
'1'	[5.4795e-05]	[0]
'S'	[0.9999]	[0]
'I'	[0]	[0.8999]
'SS'	[0]	[0]
'SI'	[-8.0000]	[8.0000]
'II'	[0]	[0]
'B*1'	[0]	[0]
'B*S'	[0]	[0]
'B*I'	[0]	[0]
'B*SS'	[0]	[0]
'B*SI'	[-2.0000]	[2.0000]
'B*II'	[0]	[0]

(b) Model Coefficients

Figure 1: Comparison of the simulated SIR model with vital dynamics, seasonal forcing, and additive noise of $\epsilon = 1 \times 10^{-10}$ with the corresponding discovered model. In this example with low relative noise, SINDy successfully identifies the correct active terms of the system, as well as the magnitude of the corresponding coefficients. $\beta_0 = 8$/wk, $\beta_1 = 0.25$, $\gamma = 0.1$/wk, $\mu = \nu = 5.4795 \times 10^{-5}$/wk.

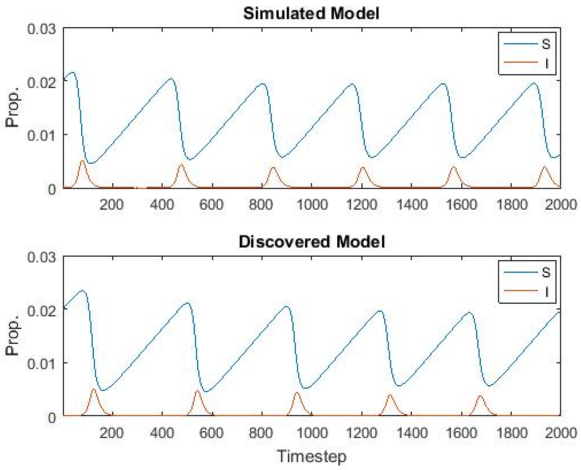

(a) Time-series

''	'Sdot'	'Idot'
'1'	5.4797e-05	0
'S'	0.9999	0
'I'	0	0.9000
'SS'	3.0604e-05	0
'SI'	-7.9999	7.9980
'II'	-5.3304e-04	0.0014
'B*1'	0	0
'B*S'	0	0
'B*I'	0	-9.9312e-06
'B*SS'	-5.3760e-06	0
'B*SI'	-2.0001	2.0021
'B*II'	4.5314e-04	-0.0013

(b) Model Coefficients

Figure 2: Comparison of the simulated SIR model with vital dynamics, seasonal forcing, and additive noise of $\epsilon = 1 \times 10^{-7}$ with the corresponding discovered model. The correct terms are still present in the discovered model, but the coefficients are no longer accurate. Other terms have also been selected in an attempt to overfit the model to the noisy data. $\beta_0 = 8/\text{wk}$, $\beta_1 = 0.25$, $\gamma = 0.1/\text{wk}$, $\mu = \nu = 5.4795 \times 10^{-5}/\text{wk}$.

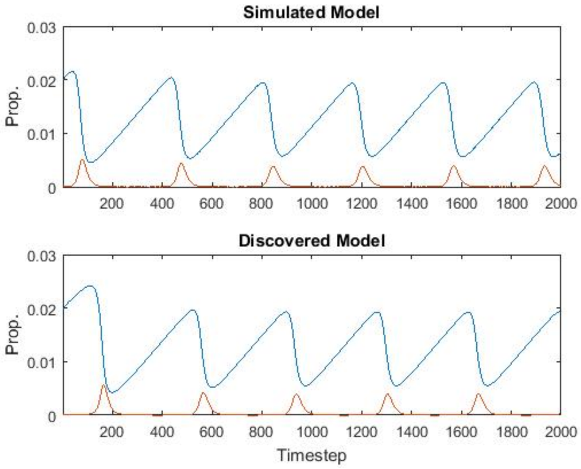

(a) Time-series

''	'Sdot'	'Idot'
'1'	5.3777e-05	2.2104e-05
'S'	1.0001	-0.0032
'I'	-9.3705e-04	0.9150
'SS'	-0.0069	0.1183
'SI'	-7.7007	0.5085
'II'	-0.4736	13.3556
'B*1'	0	-1.0606e-05
'B*S'	-1.0796e-05	0.0020
'B*I'	0.0017	-0.0232
'B*SS'	0.0011	-0.0888
'B*SI'	-2.3436	10.1537
'B*II'	0.4159	-13.0058

(b) Model Coefficients

Figure 3: Comparison of the simulated SIR model with vital dynamics, seasonal forcing, and additive noise of $\epsilon = 1 \times 10^{-5}$ with the corresponding discovered model. $\beta_0 = 8/\text{wk}$, $\beta_1 = 0.25$, $\gamma = 0.1/\text{wk}$, $\mu = \nu = 5.4795 \times 10^{-5}/\text{wk}$.

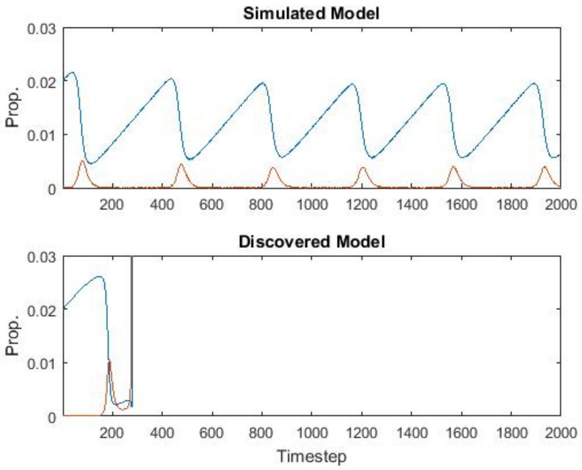

(a) Time-series

''	'Sdot'	'Idot'
'1'	[6.1967e-05]	[1.5258e-04]
'S'	[0.9990]	[-0.0206]
'I'	[-0.0154]	[0.9687]
'SS'	[0.0280]	[0.7141]
'SI'	[-6.4224]	[-40.6219]
'II'	[0.2575]	[93.5067]
'B*1'	[-1.3154e-05]	[-5.6768e-05]
'B*S'	[0.0021]	[0.0127]
'B*I'	[0.0222]	[-0.1274]
'B*SS'	[-0.0776]	[-0.5656]
'B*SI'	[-3.8226]	[54.8836]
'B*II'	[-1.4059]	[-90.6757]

(b) Model Coefficients

Figure 4: Comparison of the simulated SIR model with vital dynamics, seasonal forcing, and additive noise of $\epsilon = 5 \times 10^{-5}$ with the corresponding discovered model. $\beta_0 = 8/\mathrm{wk}$, $\beta_1 = 0.25$, $\gamma = 0.1/\mathrm{wk}$, $\mu = \nu = 5.4795 \times 10^{-5}/\mathrm{wk}$.

2 Fitting to the power spectral density

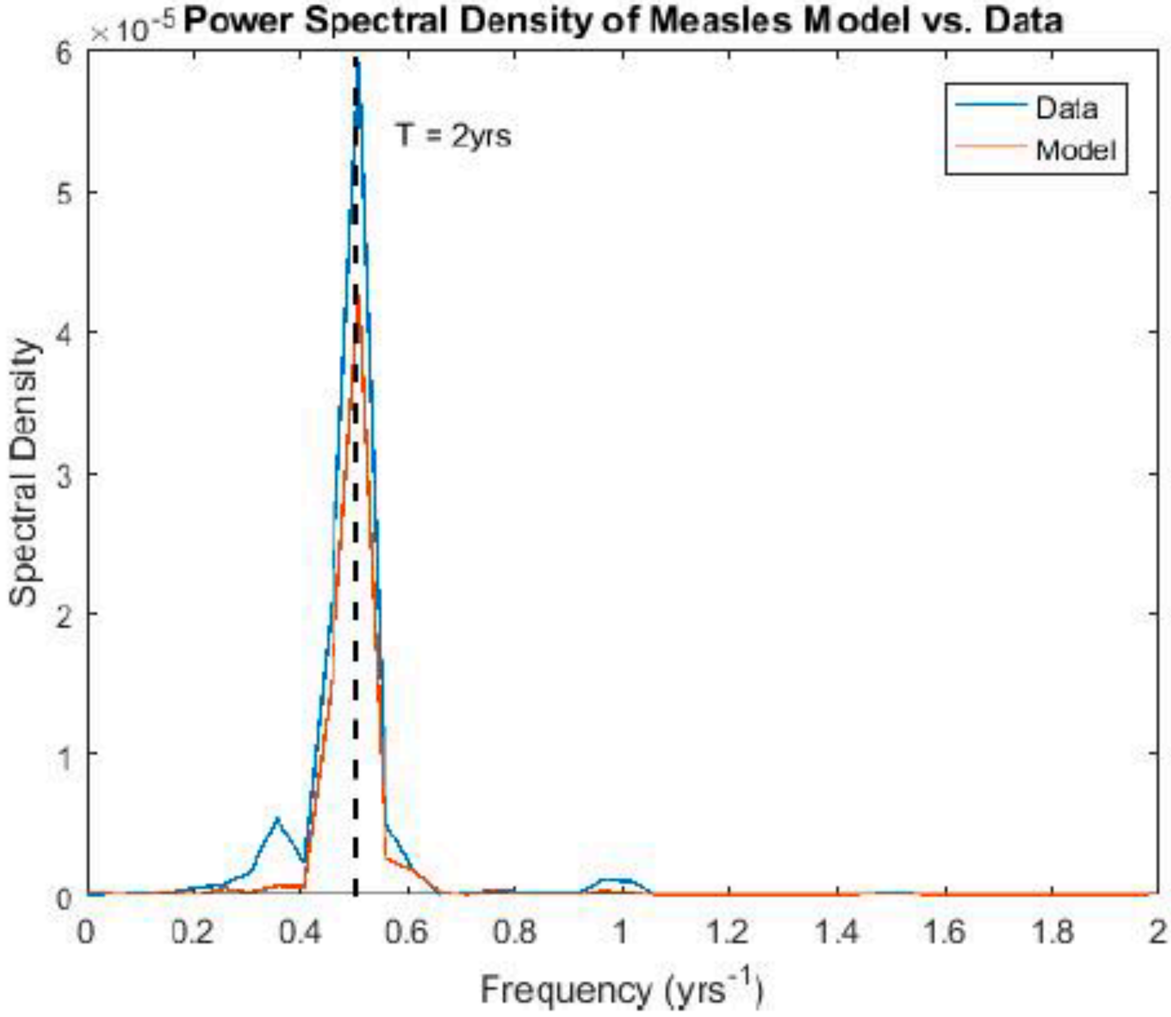

Figure 5: Comparison of power spectral density estimates of the measles data (blue) and the most parsimonious SINDy-discovered model (red). The peak corresponding to the most notable attractor present in the data (with period of 2 years) are noted with the dashed line.

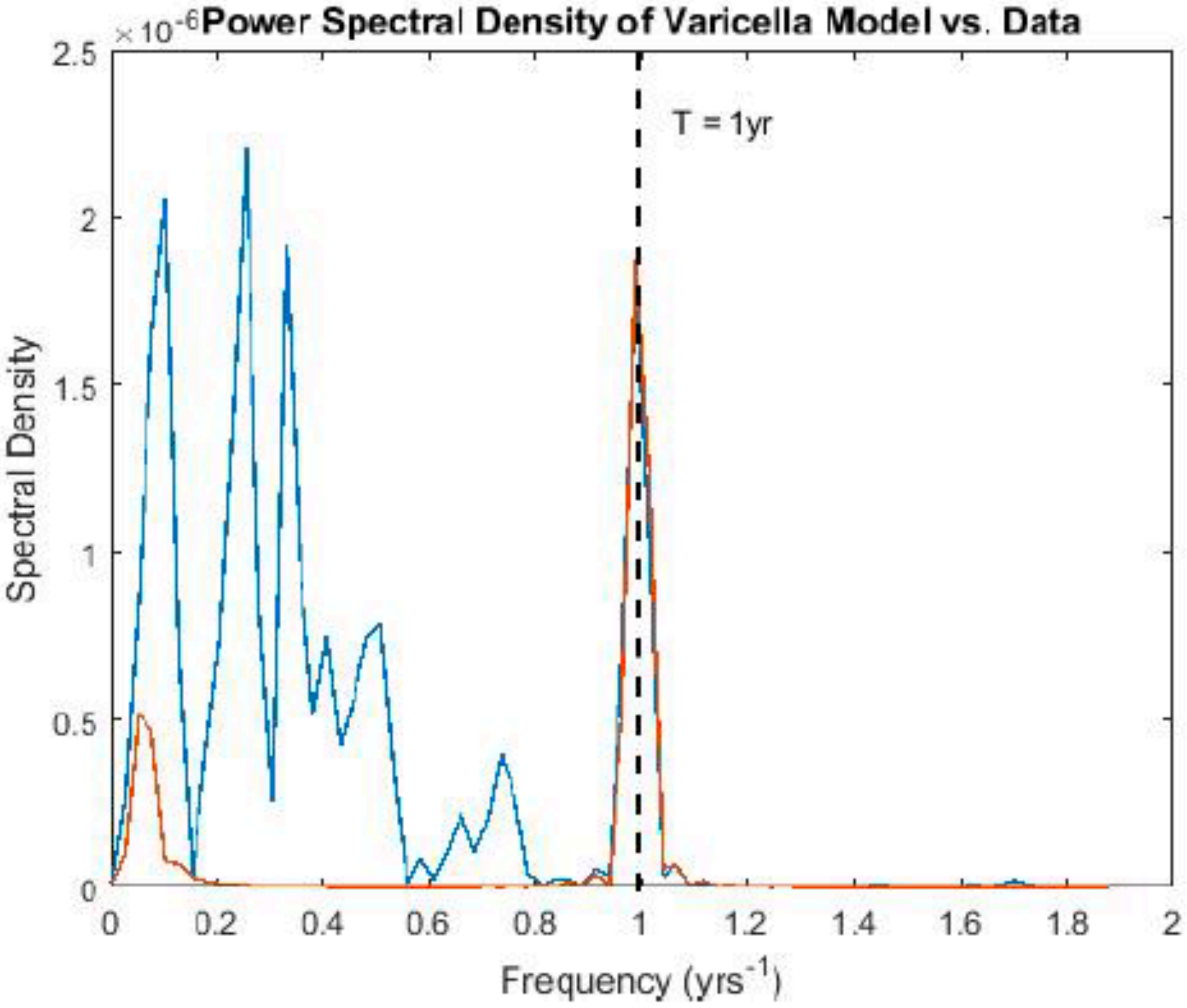

Figure 6: Comparison of power spectral density estimates of the varicella data (blue) and the most parsimonious SINDy-discovered model (red). The peak corresponding to the most notable attractor present in the data (with period of 1 year) are noted with the dashed line.

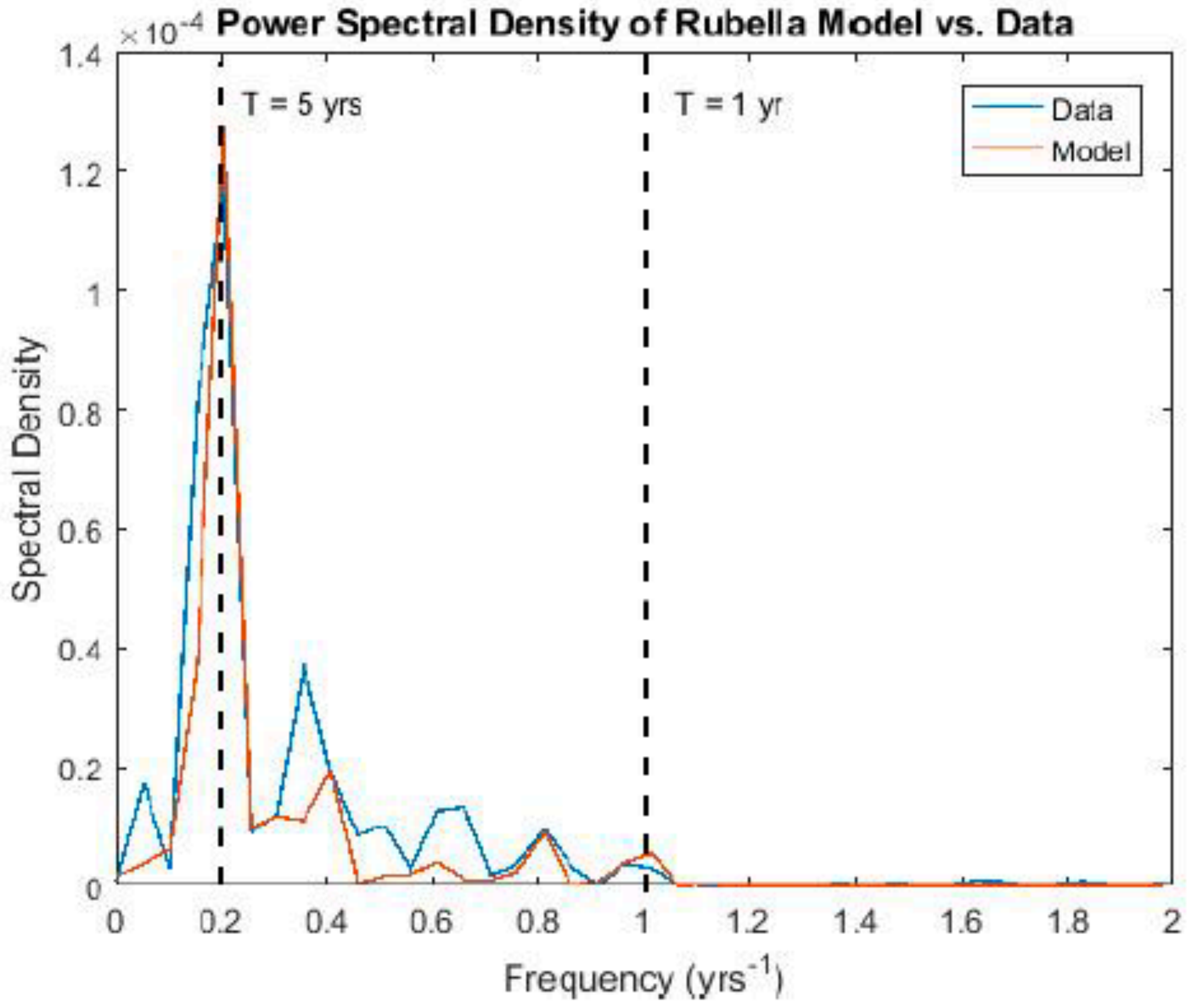

Figure 7: Comparison of power spectral density estimates of rubella data (blue) and the most parsimonious SINDy-discovered model (red). The peak corresponding to the most notable attractor present in the data (with period of 5 years) are noted with the dashed line.

3 AIC Values across $S_0 - \lambda$ parameter plane for second order libraries

3.1 Measles, parameter plane

	Initial Susceptible Values														
Lambdas	0.05	0.05571..	0.06142..	0.06714..	0.07285..	0.07857..	0.08428..	0.09	0.09571..	0.101429	0.107143	0.112857	0.118571	0.124286	0.13
0.0001	-4,538.6	-4,544.8	-4,548.1	-4,541.1	-4,548.9	-4,597.8		-4,915.3	-4,567.2	-5,044.2	-4,982.7	-5,039.7	-5,031.7	-4,968.4	-4,531.7
0.00016..	-4,538.6	-4,544.8	-4,548.1	-4,541.1	-4,548.9	-4,597.8		-4,915.3	-4,548.7	-5,044.2	-4,982.7	-5,039.7	-5,031.7	-4,968.4	-4,531.7
0.00026..	-4,538.6	-4,544.8	-4,548.1	-4,541.1	-4,548.9	-4,597.8		-4,915.3	-4,548.7	-5,044.2	-4,982.7	-5,039.7	-5,031.7	-4,968.4	-4,501.6
0.00043..	-4,538.6	-4,570.2	-4,548.1	-4,541.1	-4,548.9	-4,597.8		-5,058.2	-4,562.6	-5,044.2	-4,982.7	-5,039.7	-5,031.7	-4,968.4	-4,547.2
0.00071..	-4,544.1	-4,571.3	-4,548.1	-4,541.1	-4,548.9	-4,597.8	-4,608.1	-5,058.2	-4,545.7	-5,044.2	-4,982.7	-5,039.7	-5,031.7	-4,968.4	-4,563.6
0.00117..	-4,528.9	-4,542.4	-4,552.5	-4,561.0	-4,566.2	-4,597.8	-4,608.1	-5,058.2	-4,587.1	-5,044.2	-4,982.7	-5,039.7	-5,031.7	-4,969.5	-4,563.6
0.00193..	-4,538.8	-4,552.4	-4,563.6	-4,571.2	-4,579.1	-4,597.8	-4,608.1	-4,915.3	-4,642.5	-4,857.9	-5,063.7	-5,087.4	-5,038.3	-4,969.5	-4,589.6
0.00316..	-4,538.2	-4,552.4	-4,564.6	-4,568.2	-4,575.6	-4,775.6	-4,862.3	-4,733.9	-5,022.4	-4,857.9	-5,063.7	-5,087.4	-5,038.3	-4,799.6	-4,589.7
0.00517..	-4,560.4	-4,585.6	-4,560.2	-4,571.2	-4,580.8	-4,775.6	-4,862.3	-4,760.9	-4,945.7	-4,961.0	-5,013.9	-5,012.5	-4,999.9	-4,975.9	-4,737.8
0.00848..	-4,583.8	-4,599.8	-4,587.9	-4,539.2	-4,554.2	-4,665.3	-4,907.5	-4,862.2	-4,953.4	-4,999.5	-5,013.9	-5,012.5	-4,999.9	-4,975.9	-4,936.9
0.01389..	-4,573.1	-4,571.6	-4,564.8	-4,542.2	-4,554.2	-4,569.6	-4,578.8	-4,588.6	-5,038.2	-4,999.5	-5,027.2	-5,012.5	-4,999.9	-4,975.9	-4,936.9
0.02275..	-4,473.6	-4,612.9	-4,622.1	-4,623.2	-4,633.8	-4,610.1	-4,619.2	-4,615.2	-4,615.2	-4,600.5	-4,609.3	-4,603.5	-4,605.9	-4,963.7	-4,924.1
0.03727..	-4,355.1	-4,581.3	-4,633.5	-4,632.0	-4,633.8	-4,632.5	-4,607.4	-4,632.1	-4,614.6	-4,609.7	-4,603.9	-4,539.6	-4,506.8	-4,558.6	-4,488.0
0.06105..	-4,597.9	-4,605.4	-4,629.5	-4,606.1	-4,607.3	-4,612.1	-4,639.5	-4,639.3	-4,616.4	-4,624.7	-4,521.0	-4,505.7	-4,490.0	-4,485.8	-4,529.7
0.1	-4,634.1	-4,601.5	-4,608.9	-4,619.0	-4,636.2	-4,638.4	-4,615.9	-4,616.9	-4,542.3	-4,570.9	-4,552.1	-4,505.7	-4,499.7	-4,494.3	-4,638.9

							Initial Susceptible Values									
		0.05	0.0557	0.06142	0.06714	0.07285	0.07857	0.08428	0.09	0.09571	0.1014	0.1071	0.1129	0.1186	0.1243	0.13
	0.0001	548.8	542.6	539.3	546.3	538.5	489.6	5087.4	172.1	520.2	43.2	104.7	47.7	55.7	119	555.7
	0.00016	548.8	542.6	539.3	546.3	538.5	489.6	5087.4	172.1	538.7	43.2	104.7	47.7	55.7	119	555.7
	0.00026	548.8	542.6	539.3	546.3	538.5	489.6	5087.4	172.1	538.7	43.2	104.7	47.7	55.7	119	585.8
	0.00043	548.8	517.2	539.3	546.3	538.5	489.6	5087.4	29.2	524.8	43.2	104.7	47.7	55.7	119	540.2
	0.00071	543.3	516.1	539.3	546.3	538.5	489.6	479.3	29.2	541.7	43.2	104.7	47.7	55.7	119	523.8
	0.00117	558.5	545	534.9	526.4	521.2	489.6	479.3	29.2	500.3	43.2	104.7	47.7	55.7	117.9	523.8
lambda_s	0.00193	548.6	535	523.8	516.2	508.3	489.6	479.3	172.1	444.9	229.5	23.7	0	49.1	117.9	497.8
	0.00316	549.2	535	522.8	519.2	511.8	311.8	225.1	353.5	65	229.5	23.7	0	49.1	287.8	497.7
	0.00517	527	501.8	527.2	516.2	506.6	311.8	225.1	326.5	141.7	126.4	73.5	74.9	87.5	111.5	349.6
	0.00848	503.6	487.6	499.5	548.2	533.2	422.1	179.9	225.2	134	87.9	73.5	74.9	87.5	111.5	150.5
	0.01389	514.3	515.8	522.6	545.2	533.2	517.8	508.6	498.8	49.2	87.9	60.2	74.9	87.5	111.5	150.5
	0.02275	613.8	474.5	465.3	464.2	453.6	477.3	468.2	472.2	472.2	486.9	478.1	483.9	481.5	123.7	163.3
	0.03727	732.3	506.1	453.9	455.4	453.6	454.9	480	455.3	472.8	477.7	993.5	547.8	580.6	528.8	599.4
	0.06105	489.5	482	457.9	481.3	480.1	475.3	447.9	448.1	472.8	462.7	566.4	581.7	597.4	601.6	557.7
	0.1	453.3	485.9	478.5	468.4	451.2	449	471.5	470.5	545.1	516.5	535.3	581.7	587.7	593.1	448.5

Figure 8: Absolute (top) and relative (bottom) AIC values for SINDy models across a range of both initial susceptible and threshold values, utilizing the measles dataset and a 2nd order polynomial library. Darker colour (top) refer to a lower AIC value. Cells without value refer to a model which, when simulated, resulted in a diverging time series.

3.2 Measles, low and high sparsity threshold

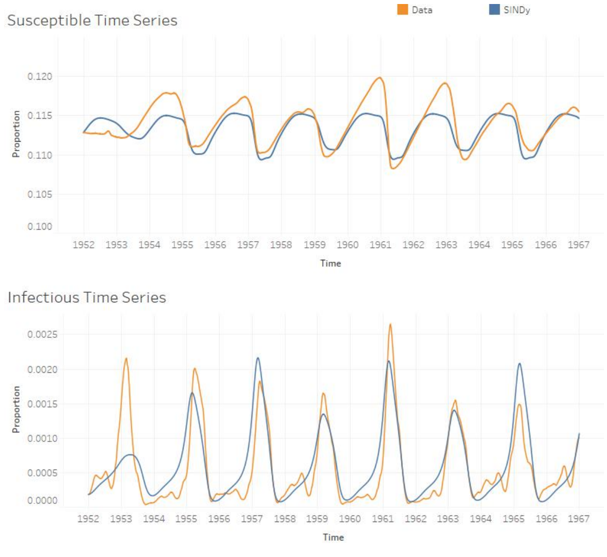

Model Coefficients

	Term											
	1	S	I	SS	SI	II	B*1	B*S	B*I	B*SS	B*SI	B*II
S Equation	0.027	0.549	-0.248	1.933	-2.665	297.915	-0.022	0.377	6.055	-1.609	-51.719	-327.798
I Equation	-0.008	0.148	-2.260	-0.664	28.080	87.865	0.000	0.003	1.711	-0.017	-14.594	-79.450

(a) $\lambda = 0.0001$.

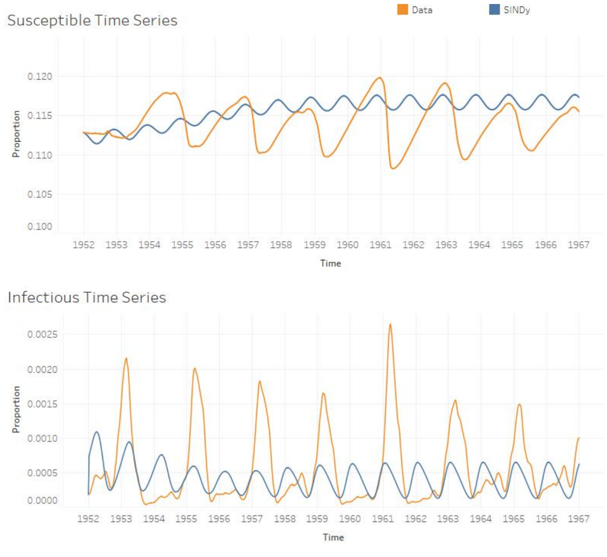

Model Coefficients

	Term											
	1	S	I	SS	SI	II	B*1	B*S	B*I	B*SS	B*SI	B*II
S Equation	-0.041	1.714	0.000	-3.125	0.000	0.000	-0.067	1.168	0.000	-5.116	0.000	0.000
I Equation	0.359	-6.213	0.000	26.876	0.000	0.000	0.392	-6.820	0.000	29.641	0.000	0.000

(b) $\lambda = 1$.

Figure 9: Resulting time series and coefficients from SINDy-discovered models of the measles dataset (with a 2nd order polynomial library) using extreme sparsity thresholds. Using a relatively small threshold ($\lambda = 0.0001$) results in a good fit and accurate recovery of attractor class, but a very low number of non-active terms, which is an indicator of an overfit model. Conversely, using a relatively large threshold ($\lambda = 1$) gives a sparse model, but at the cost of a good fit and recovery of attractor class.

3.3 Chickenpox, parameter plane

Initial Susceptible Values

Lambdas	0.05	0.05714..	0.06428..	0.07142..	0.07857..	0.08571..	0.09285..	0.1	0.107143	0.114286	0.121429	0.128571	0.135714	0.142857	0.15
0.0001	-7,220	-7,155	-7,086	-7,010	-6,925	-6,865	-6,815	-6,765	-6,735	-6,712	-6,692	-6,677	-6,665	-6,647	-6,635
0.000193070	-7,219	-7,155	-7,086	-7,010	-6,925	-6,865	-6,815	-6,765	-6,735	-6,712	-6,692	-6,677	-6,665	-6,647	-6,635
0.000372759	-7,223	-7,155	-7,088	-7,012	-6,925	-6,865	-6,815	-6,765	-6,735	-6,712	-6,692	-6,677	-6,665	-6,647	-6,635
0.000719686	-7,227	-7,161	-7,090	-7,014	-6,945	-6,880	-6,829	-6,758	-6,730	-6,716	-6,697	-6,681	-6,667	-6,649	-6,637
0.001389495	-7,227	-7,161	-7,090	-7,014	-6,940	-6,880	-6,814	-6,777	-6,725	-6,706	-6,691	-6,680	-6,664	-6,430	-6,436
0.002682696	-7,207	-7,133	-7,045	-6,948	-6,841	-6,755	-6,688	-6,641	-6,586	-6,537	-6,478	-6,450	-6,435	-6,430	-6,436
0.005179475	-7,208	-7,127	-7,032	-6,953	-6,848	-6,822	-6,774	-6,651	-6,598	-6,622	-6,512	-6,469	-6,453	-6,448	-6,451
0.01	-7,143	-7,075	-7,002	-6,953	-6,809	-6,822	-6,791	-6,606	-6,678	-6,647	-6,512	-6,677	-6,637	-6,663	-6,631
0.019306977	-7,181	-7,061	-6,915	-6,832	-6,728	-6,631	-6,706	-6,751	-6,625	-6,528	-6,385	-6,242	-6,424	-6,262	-6,370
0.037275937	-6,807	-6,725	-6,643	-6,832	-6,704	-6,672	-6,575	-6,516	-6,529	-6,496	-6,179	-6,155	94,216	-6,132	-6,370
0.071968567	-6,806	-6,722	-6,643	-6,562	-6,488	-6,419	-6,356	-6,610	-6,195	-6,203	-6,174	-6,155	-6,242	-6,225	-6,231
0.138949549	-6,750	-6,673	-6,594	-6,518	-6,447	-6,380	-6,319	-6,266	-6,146	-6,110	-6,077	-6,056	-6,043	-6,131	-6,243
0.268269580	-6,630	-6,561	-6,490	-6,421	-6,356	-6,295	-6,239	-6,189	-6,146	-6,110	-6,081	-6,060	-6,047	-6,040	-6,035
0.517947468	-6,630	-6,561	-6,490	-6,421	-6,356	-6,295	-6,239	-6,189	-6,146	-6,110	-6,081	-6,060	-6,047	-6,040	-6,040
1	-6,630	-6,561	-6,490	-6,421	-6,356	-6,295	-6,239	-6,189	-6,146	-6,110	-6,081	-6,060	-6,047	-6,040	-6,040

							Initial Susceptible Values								
		0.05	0.057	0.064	0.071	0.079	0.086	0.093	0.1	0.107	0.114	0.121	0.129	0.136	0.143	0.15
	0.0001	0	65	134	210	295	355	405	455	485	508	528	543	565	573	585
	0.00019	1	65	134	210	295	355	405	455	485	508	528	543	565	573	585
	0.00037	-3	65	132	208	295	355	405	455	485	508	528	543	565	573	585
	0.00072	-7	59	130	206	275	340	391	462	490	504	523	539	553	571	583
	0.00139	-7	59	130	206	280	340	406	443	495	514	529	540	556	790	784
	0.00268	13	87	175	272	379	465	532	579	634	683	742	770	785	790	784
lambda_s	0.00518	12	93	188	267	372	398	446	569	622	598	708	751	767	772	769
	0.01	77	145	218	267	411	398	429	614	542	573	708	543	583	557	589
	0.019	39	159	305	388	492	589	514	469	595	692	835	978	796	958	850
	0.037	413	495	577	388	516	548	645	704	691	724	1041	1065	-86996	1088	850
	0.072	414	498	577	658	732	801	864	610	1025	1017	1046	1065	978	995	989
	0.139	470	547	626	702	773	840	901	954	1074	1110	1143	1164	1177	1089	977
	0.268	590	659	730	799	864	925	981	1031	1074	1110	1139	1160	1173	1180	1185
	0.518	590	659	730	799	864	925	981	1031	1074	1110	1139	1160	1173	1180	1180
	0.1	590	659	730	799	864	925	981	1031	1074	1110	1139	1160	1173	1180	1180

Figure 10: Absolute (top) and relative (bottom) AIC values for SINDy models across a range of both initial susceptible and threshold values, utilizing the chickenpox dataset and a 2nd order polynomial library. Darker colour refers to a lower AIC value. Cells without value refer to a model which, when simulated, resulted in a diverging time series.

3.4 Chickenpox, low sparsity threshold

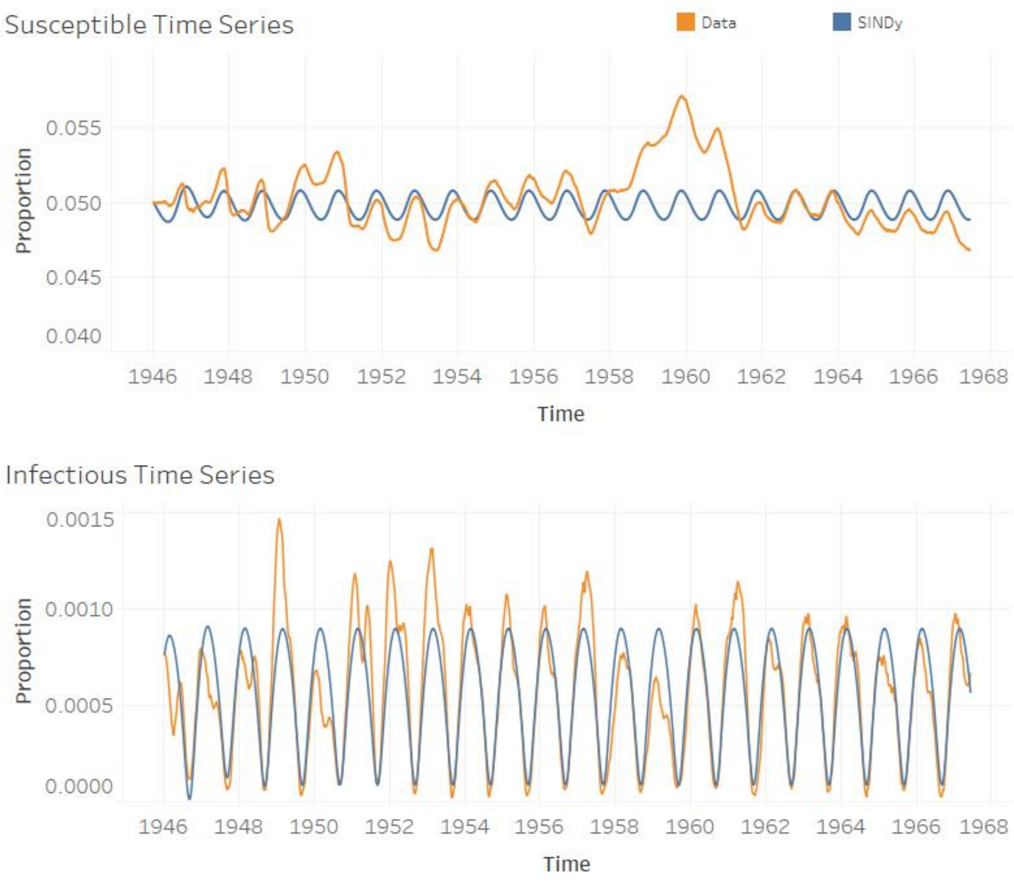

Model Coefficients

Term	S Equat..	I Equati..
1	0.000	-0.002
S	1.017	0.077
I	1.060	0.863
SS	-0.097	-0.747
SI	-27.062	1.224
II	11.284	19.891
B*1	0.000	-0.001
B*S	0.019	0.007
B*I	-0.371	1.149
B*SS	-0.238	0.023
B*SI	6.077	-19.005
B*II	32.865	-108.661

Figure 11: Resulting time series and coefficients from SINDy-discovered models of the chickenpox dataset (with a 2nd order polynomial library) using a relatively low threshold ($\lambda = 0.0001$). The resulting model exhibits a good fit and accurate recovery of attractor class, but a very low number of non-active terms, which is an indicator of an overfit model.

3.5 Chickenpox, high sparsity threshold

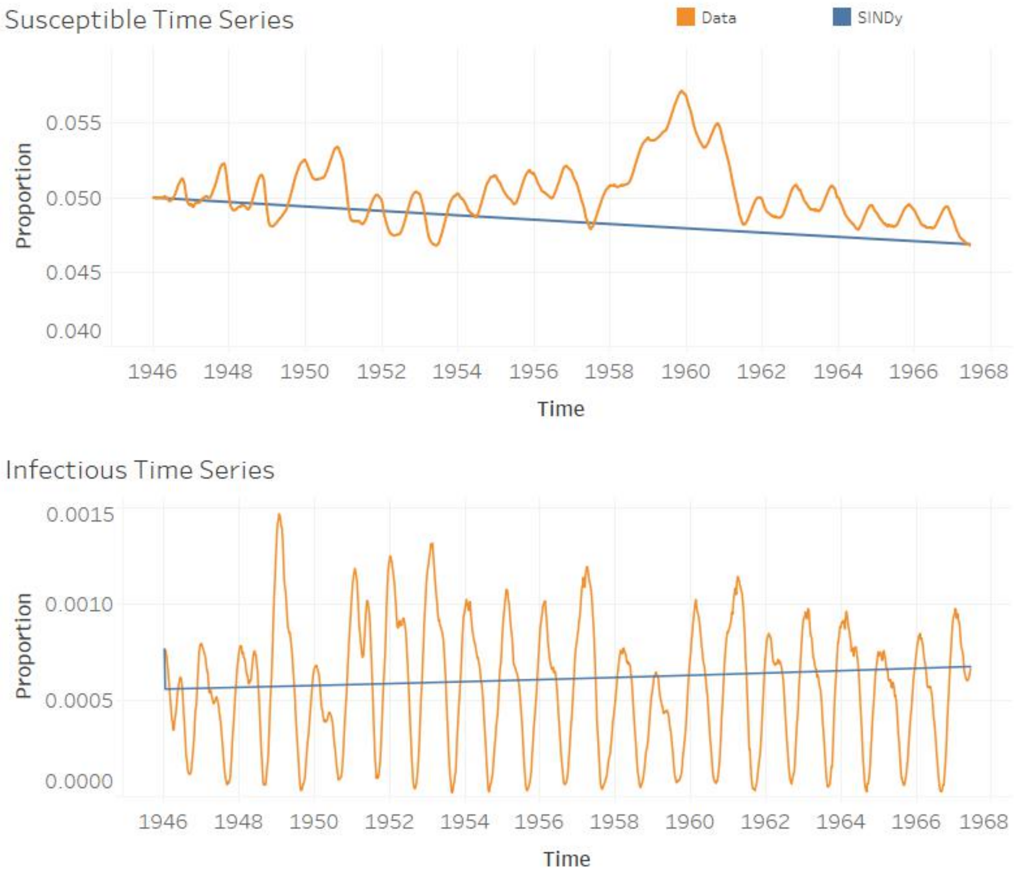

Model Coefficients

Term	S Equat..	I Equati..
1	0.000	0.008
S	1.000	-0.286
I	0.000	0.000
SS	0.000	2.567
SI	0.000	0.000
II	0.000	0.000
B*1	0.000	0.000
B*S	0.000	0.000
B*I	0.000	0.000
B*SS	0.000	0.000
B*SI	0.000	0.000
B*II	0.000	0.000

Figure 12: Resulting time series and coefficients from SINDy-discovered models of the chickenpox dataset (with a 2nd order polynomial library) using a relatively high threshold ($\lambda = 1$). The resulting model exhibits a high level of sparsity, but the linear fit does not accurately represent the dynamics of the system whatsoever.

3.6 Rubella, parameter plane

	Initial Susceptible Values														
Lambdas	0.03	0.03714..	0.04428..	0.05142..	0.05857..	0.06571..	0.07285..	0.08	0.08714..	0.09428..	0.101429	0.108571	0.115714	0.122857	0.13
0.0001	-3,465	-3,468	-3,469	-3,491	-3,527	-3,575	-3,631	-3,667	-3,718	-3,745	-3,770	-3,792	-3,810	-3,817	-3,792
0.000193070	-3,456	-3,468	-3,481	-3,504	-3,527	-3,575	-3,631	-3,667	-3,718	-3,745	-3,770	-3,792	-3,810	-3,817	-3,792
0.000372759	-3,465	-3,474	-3,481	-3,522	-3,562	-3,590	-3,651	-3,705	-3,752	-3,784	-3,770	-3,792	-3,810	-3,817	-3,792
0.000719686	-3,451	-3,485	-3,490	-3,512	-3,542	-3,585	-3,640	-3,694	-3,718	-3,745	-3,770	-3,793	-3,812	-3,823	-3,799
0.001389495	-3,641	-3,497	-3,499	-3,517	-3,542	-3,585	-3,647	-3,694	-3,718	-3,745	-3,770	-3,817	-3,840	-3,843	-3,817
0.002682696	-3,532	-3,411	-3,418	-3,579	-3,348	-3,535	-3,596	-3,653	-3,693	-3,734	-3,770	-3,797	-3,816	-3,813	-3,787
0.005179475	-1,189	-3,039	-3,362	-3,384	-3,409	-3,441	-3,468	-3,560	-3,599	-3,611	-3,677	-3,741	-3,779	-3,793	-3,791
0.01	-1,189	-1,399	-1,564	-3,460	-3,509	-3,385	-3,846	-3,713	-3,814	-3,851	-3,797	-3,688	-3,809	-3,831	-3,839
0.019306977	-3,579	-3,587	-1,564	-3,619	-3,542	-3,590	-3,590	-3,664	-3,734	-3,800	-3,791	-3,818	-3,809	-3,832	-3,818
0.037275937	-3,579	-3,587	-3,602	-3,619	-3,637	-3,645	-3,664	-3,681	-3,746	-3,713	-3,726	-3,739	-3,751	-3,767	-3,775
0.071968567	-3,579	-3,587	-3,602	-3,619	-3,637	-3,654	-3,670	-3,686	-3,701	-3,714	-3,723	-3,737	-3,745	-3,758	-3,775
0.138949549	-3,579	-3,587	-3,602	-3,619	-3,637	-3,654	-3,670	-3,686	-3,701	-3,714	-3,727	-3,740	-3,751	-3,762	-3,773
0.268269580	-3,579	-3,587	-3,602	-3,619	-3,637	-3,654	-3,670	-3,686	-3,701	-3,714	-3,727	-3,740	-3,751	-3,762	-3,772
0.517947468	-3,579	-3,587	-3,602	-3,619	-3,637	-3,654	-3,670	-3,686	-3,701	-3,714	-3,727	-3,740	-3,751	-3,762	-3,772
1	-3,579	-3,587	-3,602	-3,619	-3,637	-3,654	-3,670	-3,686	-3,701	-3,714	-3,727	-3,740	-3,751	-3,762	-3,772

								Initial Susceptible Values								
		0.03	0.037	0.044	0.051	0.059	0.066	0.073	0.08	0.087	0.094	0.101	0.109	0.116	0.123	0.13
	0.0001	381	378	377	355	319	271	215	179	128	101	76	54	36	29	54
	0.00019	390	378	365	342	319	271	215	179	128	101	76	54	36	29	54
	0.00037	381	372	365	324	284	256	195	141	94	62	76	54	36	29	54
	0.00072	395	361	356	334	304	261	206	152	128	101	76	53	34	23	47
	0.00139	205	349	347	329	304	261	199	152	128	101	76	29	6	3	29
	0.00268	314	435	428	267	498	311	250	193	153	112	76	49	30	33	59
lambda_s	0.00518	2657	807	484	462	437	405	378	286	247	235	169	105	67	53	55
	0.01	2657	2447	2282	386	337	461	0	133	32	-5	49	158	37	15	7
	0.019	267	259	2282	227	304	256	256	182	112	46	55	28	37	14	28
	0.037	267	259	244	227	209	201	182	165	100	133	120	107	95	79	71
	0.072	267	259	244	227	209	192	176	160	145	132	123	109	101	88	71
	0.139	267	259	244	227	209	192	176	160	145	132	119	106	95	84	113
	0.268	267	259	244	227	209	192	176	160	145	132	119	106	95	84	74
	0.518	267	259	244	227	209	192	176	160	145	132	119	106	95	84	74
	0.1	267	259	244	227	209	192	176	160	145	132	119	106	95	84	74

Figure 13: Absolute (top) and relative (bottom) AIC values for SINDy models across a range of both initial susceptible and threshold values, utilizing the rubella dataset and a 2nd order polynomial library. Darker colour refers to a lower AIC value. Cells without value refer to a model which, when simulated, resulted in a diverging time series.

3.7 Rubella, low sparsity threshold

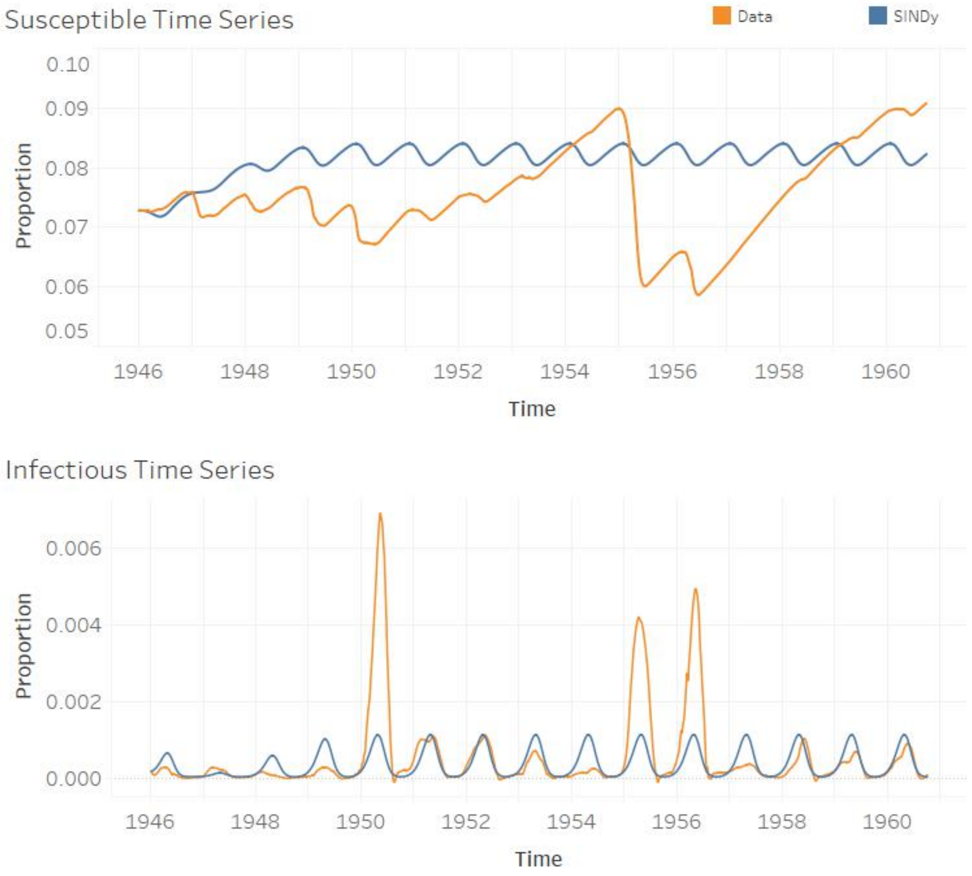

Model Coefficients

Term	S Equation	I Equation
1	0.004	0.001
S	0.891	-0.023
I	-0.037	0.886
SS	0.717	0.154
SI	-3.393	0.176
II	27.644	11.455
B*1	0.003	0.001
B*S	-0.091	-0.034
B*I	1.220	0.573
B*SS	0.632	0.228
B*SI	-16.649	-4.791
B*II	-41.412	-6.796

Figure 14: Resulting time series and coefficients from SINDy-discovered models of the rubella dataset (with a 2nd order polynomial library) using a relatively low threshold ($\lambda = 0.0001$). The resulting model exhibits neither sparsity nor a well-fitting time series, indicating there is no benefit decreasing the sparsity threshold.

3.8 Rubella, high sparsity threshold

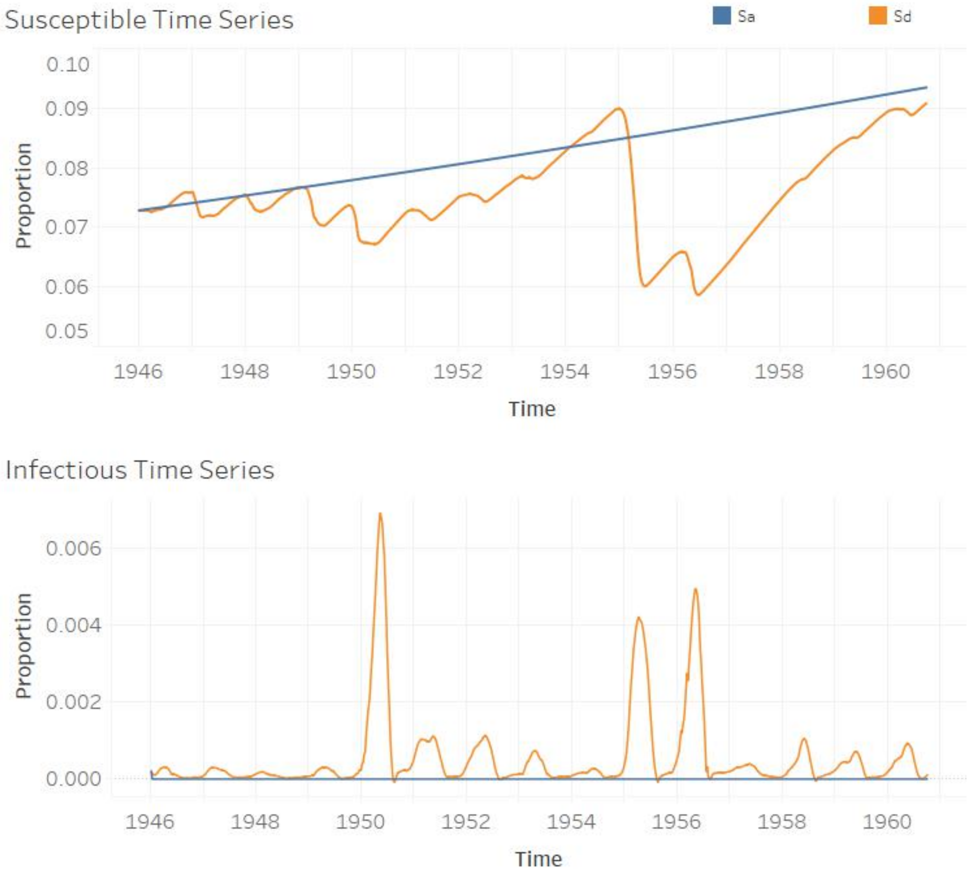

Model Coefficients

Term	S Equation	I Equation
1	0.000	0.000
S	1.000	0.000
I	0.000	0.000
SS	0.000	0.000
SI	0.000	0.000
II	0.000	0.000
B*1	0.000	0.000
B*S	0.000	0.000
B*I	0.000	0.000
B*SS	0.000	0.000
B*SI	0.000	0.000
B*II	0.000	0.000

Figure 15: Resulting time series and coefficients from SINDy-discovered models of the rubella dataset (with a 2nd order polynomial library) using a relatively high threshold ($\lambda = 1$). Once the threshold is increased past a critical value, the model is reduced to a linear, which is certainly sparse but does not match the dynamics of the system.

4 Results using third-order library

4.1 Measles

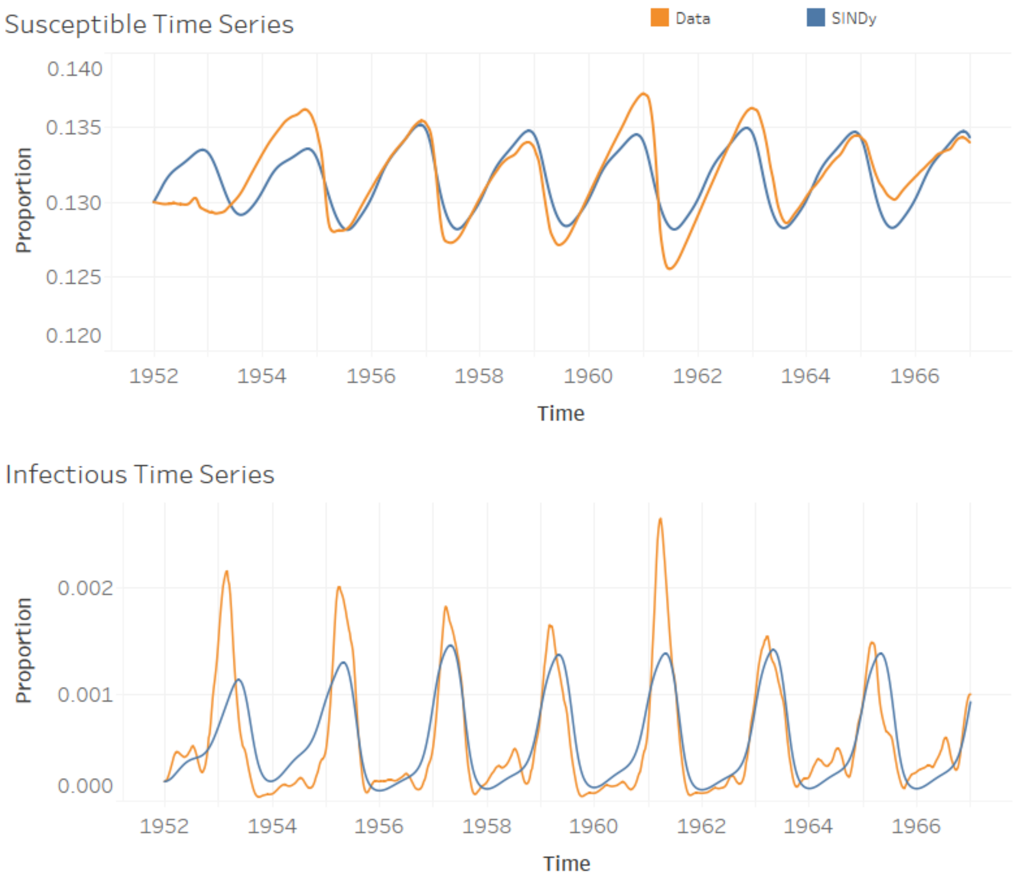

Model Coefficients

Term	S Eq.	I Eq.
1	0.269	-0.297
S	-7.495	9.159
I	90.227	-9.872
SS	89.386	-94.257
SI	-1867.728	210.076
II	0.000	0.000
SSS	-313.471	323.266
SSI	9627.518	-1007.408
SII	0.000	0.000
III	0.000	0.000
B*1	0.013	0.167
B*S	0.000	-5.145
B*I	-72.365	-31.363
B*SS	-3.996	52.888
B*SI	1456.711	638.044
B*II	0.000	0.000
B*SSS	27.338	-181.174
B*SSI	-7316.794	-3238.079
B*SII	0.000	0.000
B*III	0.000	0.000

Figure 16: Comparison between measles incidence data and the best SINDy-discovered model using a function library of polynomials up to 3rd order. As in the case above, the discovered model accurately replicates the biennium present in the data in both the susceptible and infection classes. It also again identifies a strong dependence on the SI cross term, as well as the S^2I and (to a lesser extent) the S^3 terms. The sparse regression resulted in the exclusion of thirteen terms, giving a regularization ratio of 0.325.

4.2 Chickenpox

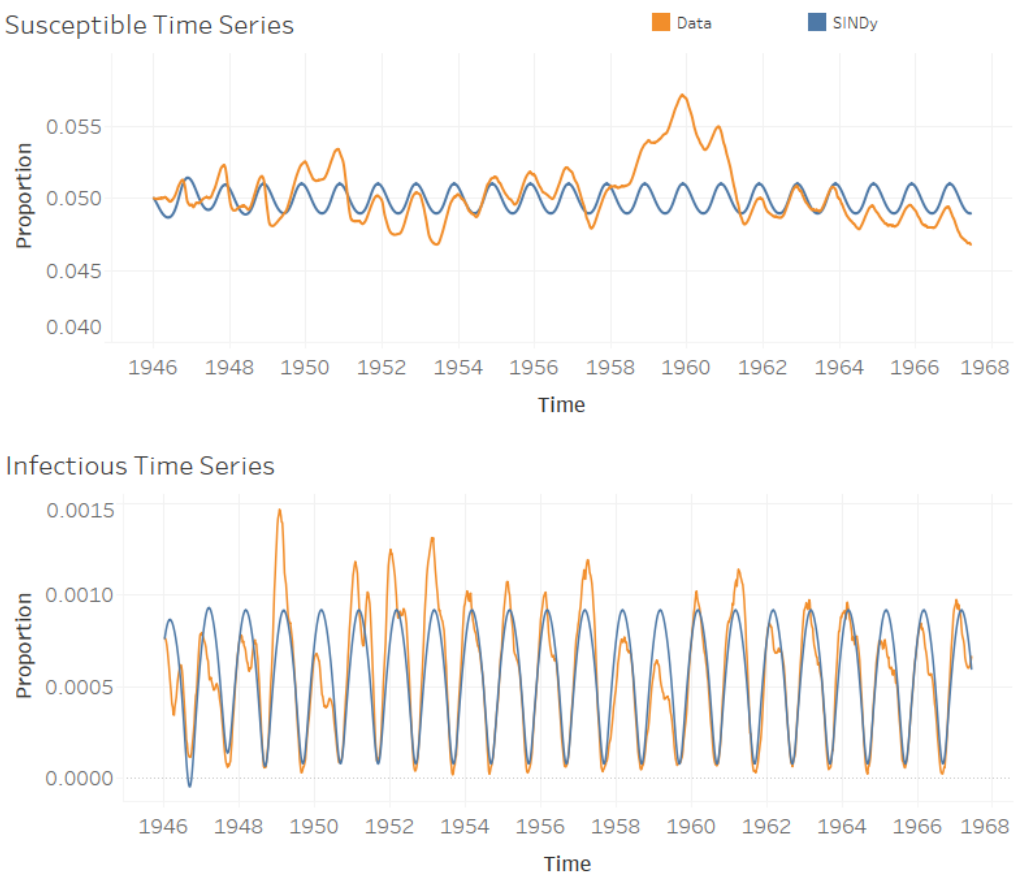

Model Coefficients

Term	S Eq.	I Eq.
1	-0.085	0.017
S	5.814	-0.953
I	12.727	-9.035
SS	-90.806	17.519
SI	-525.396	368.899
II	1935.156	852.312
SSS	569.506	-107.465
SSI	5295.210	-3411.586
SII	-38560.313	-16089.131
III	0.000	0.000
B*1	0.004	0.028
B*S	-0.114	-1.729
B*I	0.000	5.587
B*SS	-0.137	35.611
B*SI	0.000	-193.224
B*II	0.000	390.591
B*SSS	16.198	-241.889
B*SSI	0.000	1717.189
B*SII	0.000	-10214.934
B*III	0.000	0.000

Figure 17: Comparison between chickenpox incidence data and the best SINDy-discovered model using a function library of polynomials up to 3rd order. Again the discovered model accurately replicates the annual cycle present in the data in both the susceptible and infection classes. The dependence on the mass action incidence term is again noticeable, though the S^2I, SI^2 and S^3 terms have dominant coefficients as well. The sparse regression resulted in the exclusion of nine terms, giving a regularization ratio of 0.225.

4.3 Rubella

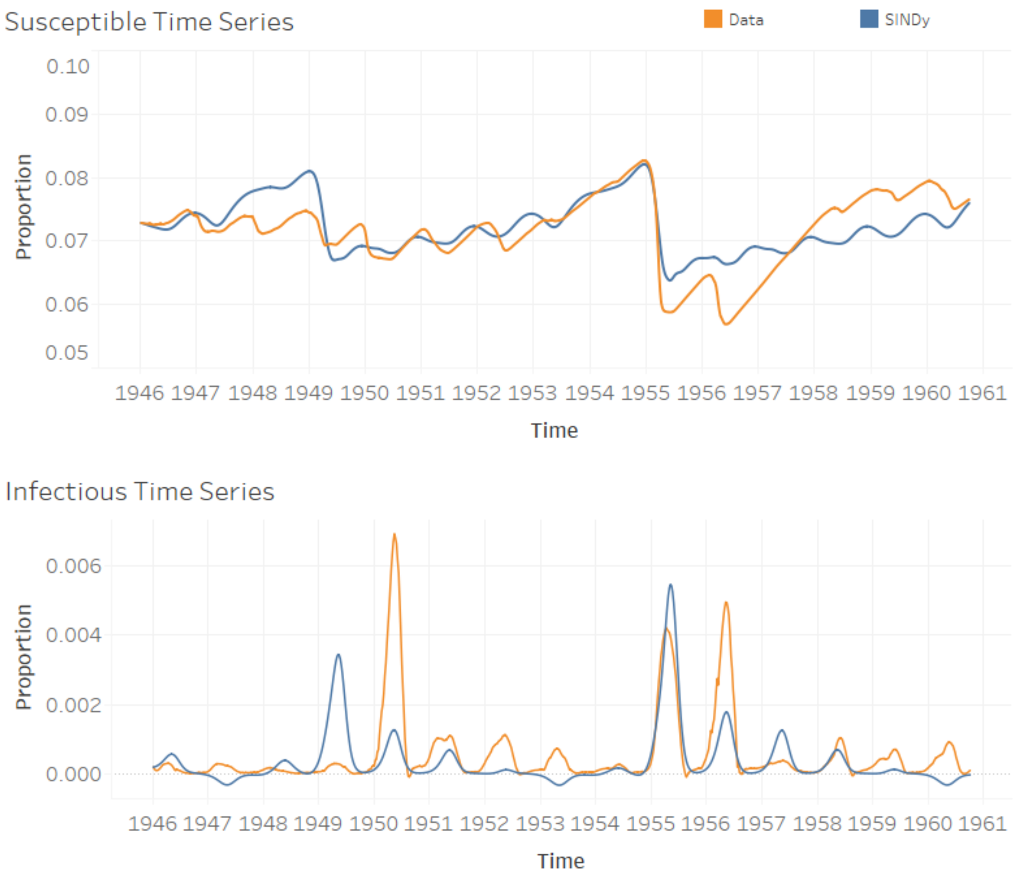

Model Coefficients

Term	S Eq.	I Eq.
1	0.029	-0.024
S	-0.083	1.039
I	-6.752	1.638
SS	13.444	-14.652
SI	211.781	-21.401
II	-64.610	0.000
SSS	-54.608	68.677
SSI	-1658.577	165.223
SII	0.000	0.000
III	9702.692	0.000
B*1	0.039	-0.027
B*S	-1.540	1.172
B*I	-1.944	1.273
B*SS	19.968	-16.644
B*SI	75.604	-22.778
B*II	-373.355	-144.183
B*SSS	-85.225	78.579
B*SSI	-589.281	98.566
B*SII	0.000	2200.994
B*III	48493.437	0.000

Figure 18: Comparison between rubella incidence data and the best SINDy-discovered model using a function library of polynomials up to 3rd order. The discovered model successfully recovers a multi-annual cycle in the prevalence time series, similar to the one present in the data. It also replicates the temporal fluctuations from the mean present in the susceptible reconstruction. The dependence on the mass action incidence term again exists, though perhaps not as strong as in models recovered from the other diseases. The sparse regression resulted in the exclusion of six terms, giving a regularization ratio of 0.15.

4.4 AIC Values across $S_0 - \lambda$ parameter plane for third order libraries

4.4.1 Measles, parameter plane

Initial Susceptible Values

Lambdas	0.03	0.03714..	0.04428..	0.05142..	0.05857..	0.06571..	0.07285..	0.08	0.08714..	0.09428..	0.101429	0.108571	0.115714	0.122857	0.13
0.0001	-4,516	-4,537	-4,704	-4,229	-4,835	-4,909		-4,273		-4,937	-4,509	-4,489		-5,047	-5,059
0.000193070	-4,516	-4,537	-4,704	-4,229	-4,835	-4,909	-4,418	-4,273		-4,937	-4,509	-4,489	-4,941	-5,047	-5,059
0.000372759	-4,524	-4,537	-4,706	-4,480	-4,561	-4,909	-4,418	-4,275		-4,937	-4,509	-4,489	-5,010	-5,047	-5,046
0.000719686	-4,528	-4,457	-4,706	-4,506	-4,848	-4,549		-4,489		-4,589	-4,509	-4,489	-5,010	-5,047	-4,989
0.001389495	-4,597	-4,537	-4,640		-4,848	-4,451	-4,870	-4,815		-4,589	-4,511	-4,821	-4,504	-5,049	-4,988
0.002682696	-4,560	-4,526	-4,570	-4,377	-4,551	-4,523	-4,870	-4,815	-4,853	-4,686	-4,633	-4,521	-4,767	-4,597	
0.005179475	-4,641	-4,416	-4,617	-4,525	-4,576	-4,321	-4,870	-4,701	-4,853	-4,686	-4,862	-4,953	-4,638	-4,935	-4,960
0.01	-4,629	-4,519	-4,585	-4,603	-4,779	-4,669	-4,817	-4,838	-4,853	-4,686	-4,633	-4,538	-4,330	-4,935	-4,883
0.019306977	-4,639	-4,521	-4,607	-4,664	-4,658	-4,752	-4,955	-4,542		-4,703	-4,643	-4,816	-4,967	-5,037	-5,070
0.037275937	-4,671	-4,592	-4,607	-4,871	-4,757	-4,901	-4,833	-4,565	-4,845	-4,753	-4,872	-4,925	-5,004	-5,110	-5,035
0.071968567	-4,670	-4,670	-4,665	-4,487	-4,483	-4,549	-4,632	-4,840	-4,693	-4,879	-4,770	-4,925	-5,004	-5,110	-5,035
0.138949549	-4,695	-4,672	-4,423	-4,616	-4,629	-4,612	-4,632	-4,840	-4,536	-4,572	-4,795	-4,794	-4,851	-4,957	-4,883
0.268269580	-4,680	-4,678	-4,656	-4,647	-4,641	-4,619	-4,611	-4,540	-4,622	-4,574	-4,567	-4,551	-4,524	-4,614	-4,617
0.517947468	-4,678	-4,679	-4,675	-4,515	-4,660	-4,612	-4,629	-4,537	-4,563	-4,572	-4,567	-4,551	-4,537	-4,624	-4,598
1	-2,657	-4,679	-4,675	-4,668	-4,660	-4,653	-4,633	-4,629	-4,584	-4,575	-4,589	-4,590	-4,627	-4,614	-4,617

Figure 19: The AIC values for SINDy models across a range of both initial susceptible and threshold values, utilizing the measles dataset and a 3rd order polynomial library. Darker colour refers to a lower AIC value. Cells without value refer to a model which, when simulated, resulted in a diverging time series.

4.4.2 Measles, low sparsity threshold

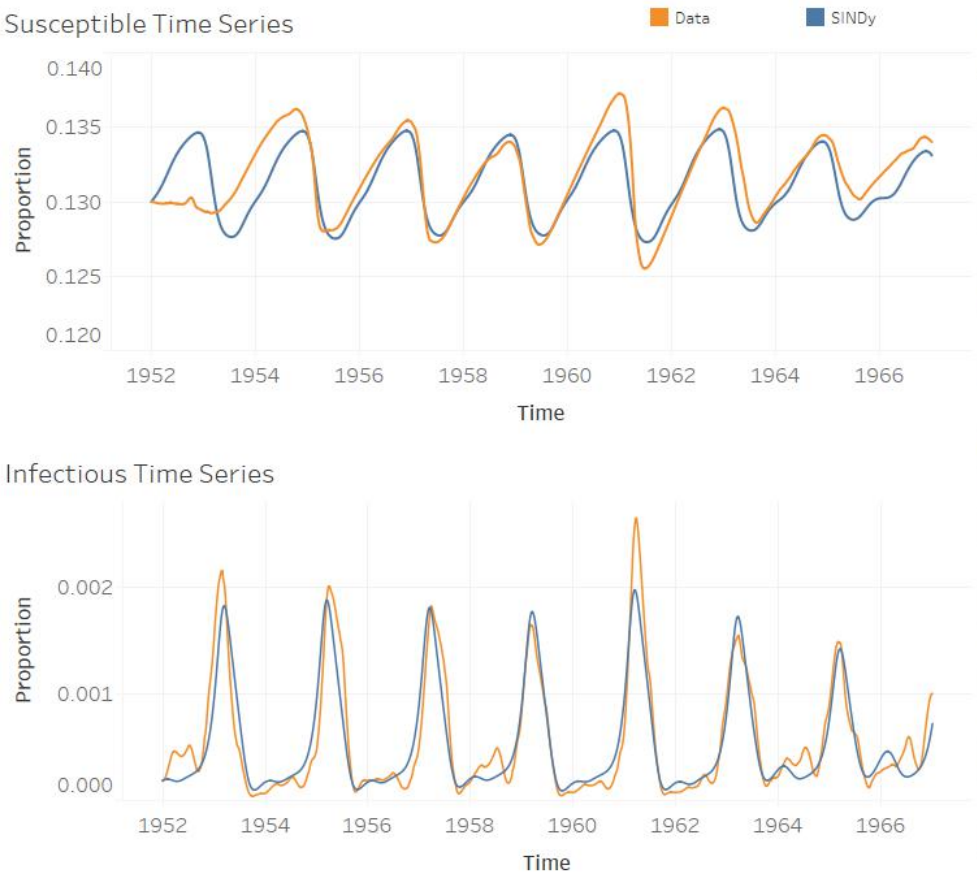

Model Coefficients

Term	S Equation	I Equation
1	0.305	-0.510
S	-6.833	11.400
I	351.061	-5.452
SS	66.203	-84.899
SI	-5327.468	96.382
II	2869.712	-442.824
SSS	-184.485	210.739
SSI	20186.514	-360.189
SII	-22012.324	3673.821
III	30879.409	-5619.322
B*1	3.678	-0.396
B*S	-83.204	9.371
B*I	98.682	-69.649
B*SS	627.442	-73.775
B*SI	-1542.972	1050.404
B*II	4334.223	-379.699
B*SSS	-1577.114	193.204
B*SSI	6031.372	-3957.069
B*SII	-34558.637	3153.581
B*III	120009.452	0.000

Figure 20: Resulting time series and coefficients from SINDy-discovered models of the measles dataset (with a 3rd order polynomial library) using a relatively low threshold ($\lambda = 0.0001$). The resulting model exhibits a good fit and accurate recovery of attractor class, but a very low number of non-active terms, which is an indicator of an overfit model.

4.4.3 Measles, high sparsity threshold

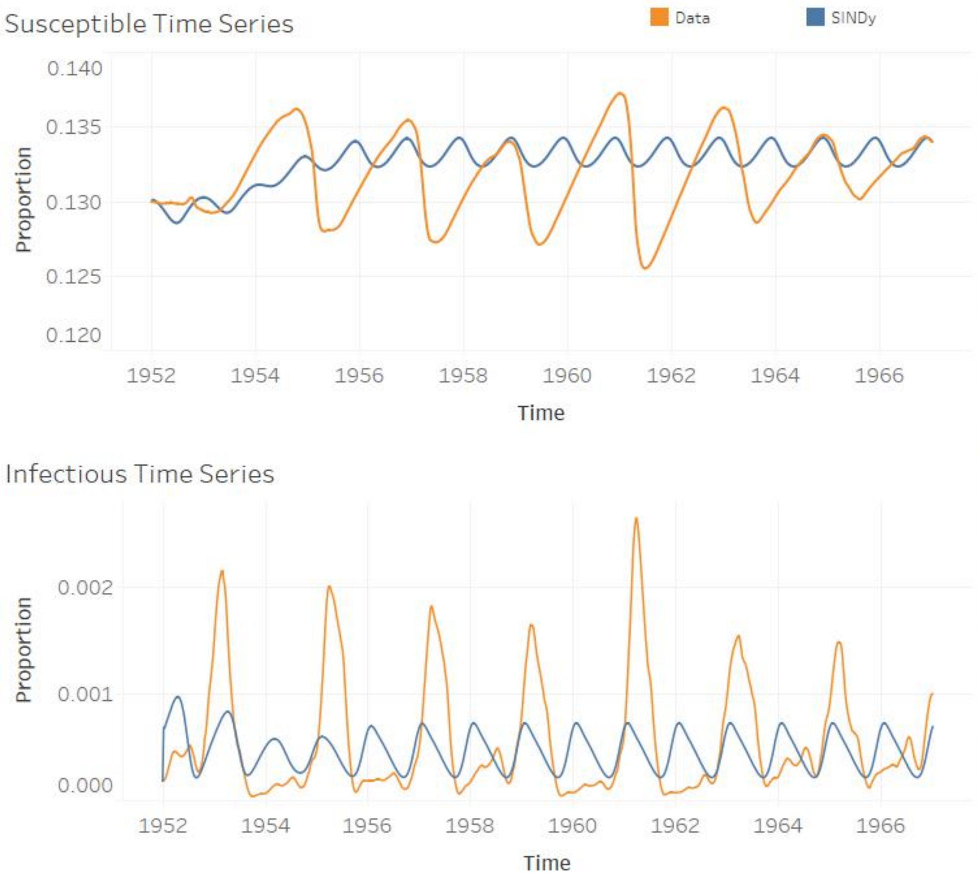

Model Coefficients

Term	S Equation	I Equation
1	-2.199	9.658
S	50.156	-216.002
I	213.931	0.000
SS	-366.080	1609.843
SI	-3249.802	0.000
II	0.000	0.000
SSS	908.250	-3998.005
SSI	12323.463	0.000
SII	0.000	0.000
III	0.000	0.000
B*1	3.136	-7.118
B*S	-70.539	156.556
B*I	-93.657	0.000
B*SS	528.734	-1146.285
B*SI	1393.328	0.000
B*II	0.000	0.000
B*SSS	-1320.754	2793.806
B*SSI	-5174.140	0.000
B*SII	0.000	0.000
B*III	0.000	0.000

Figure 21: Resulting time series and coefficients from SINDy-discovered models of the measles dataset (with a 3rd order polynomial library) using a relatively high threshold ($\lambda = 1$). The resulting model exhibits sparsity, but at the cost of a good fit and recovery of attractor class.

4.4.4 Chickenpox, parameter plane

Initial Susceptible Values

Lambdas	0.05	0.05714..	0.06428..	0.07142..	0.07857..	0.08571..	0.09285..	0.1	0.107143	0.114286	0.121429	0.128571	0.135714	0.142857	0.15
0.0001	-7,167	-7,094	-7,039	-6,977	-6,911	-6,850	-6,790	-6,742	-6,705	-6,678	-6,655	-6,638	-6,623	-6,609	-6,597
0.000193070	-7,167	-7,099	-7,039	-6,977	-6,911	-6,850	-6,790	-6,742	-6,705	-6,678	-6,655	-6,638	-6,623	-6,609	-6,597
0.000372759	-7,168	-7,099	-7,043	-6,982	-6,918	-6,858	-6,790	-6,742	-6,707	-6,680	-6,655	-6,638	-6,623	-6,609	-6,597
0.000719686	-7,168	-7,099	-7,043	-6,982	-6,918	-6,858	-6,798	-6,751	-6,715	-6,679	-6,657	-6,637	-6,622	-6,609	-6,597
0.001389495	-7,168	-7,099	-7,043	-6,987	-6,919	-6,852	-6,793	-6,746	-6,711	-6,686	-6,666	-6,641	-6,629	-6,621	-6,614
0.002682696	-7,168	-7,116	-7,063	-6,988	-6,919	-6,852	-6,793	-6,746	-6,711	-6,688	-6,666	-6,647	-6,636	-6,621	-6,614
0.005179475	-7,191	-7,118	-7,021	-6,992	-6,919	-6,852	-6,793	-6,746	-6,713	-6,690	-6,669	-6,647	-6,637	-6,629	-6,622
0.01	-7,163	-7,100	-7,031	-6,932	-6,922	-6,851	-6,793	-6,750	-6,716	-6,690	-6,669	-6,651	-6,637	-6,629	-6,622
0.019306977	-7,197	-7,107	-7,031	-6,941	-6,846	-6,793	-6,804	-6,757	-6,722	-6,692	-6,669	-6,654	-6,643	-6,633	-6,628
0.037275937	-7,162	-7,107	-7,031	-6,941	-6,854	-6,796	-6,762	-6,727	-6,701	-6,679	-6,664	-6,652	-6,639	-6,630	-6,627
0.071968567	-7,165	-7,086	-7,039	-6,942	-6,858	-6,799	-6,762	-6,727	-6,701	-6,679	-6,664	-6,652	-6,639	-6,630	-6,624
0.138949549	-7,179	-7,090	-6,910	-6,840	-6,854	-6,793	-6,743	-6,723	-6,691	-6,679	-6,664	-6,652	-6,639	-6,630	-6,624
0.268269580	-7,179	-7,088	-6,967	-6,807	-6,639	-6,684	-6,608	-6,517	-6,440	-6,588	-6,610	-6,628	-6,630	-6,630	-6,630
0.517947468	-6,406	-6,439	-6,436	-6,807	-6,639	-6,604	-6,543	-6,449	-6,374	-6,313	-6,265	-6,230	-6,203	-6,194	-6,500
1	-6,383	-6,406	-6,620	-6,697	-6,703	-6,480	-6,362	-6,449	-6,374	-6,313	-6,265	-6,230	-6,205	-6,194	-6,183

Figure 22: The AIC values for SINDy models across a range of both initial susceptible and threshold values, utilizing the chickenpox dataset and a 3rd order polynomial library. Darker colour refers to a lower AIC value. Cells without value refer to a model which, when simulated, resulted in a diverging time series.

4.4.5 Chickenpox, low sparsity threshold

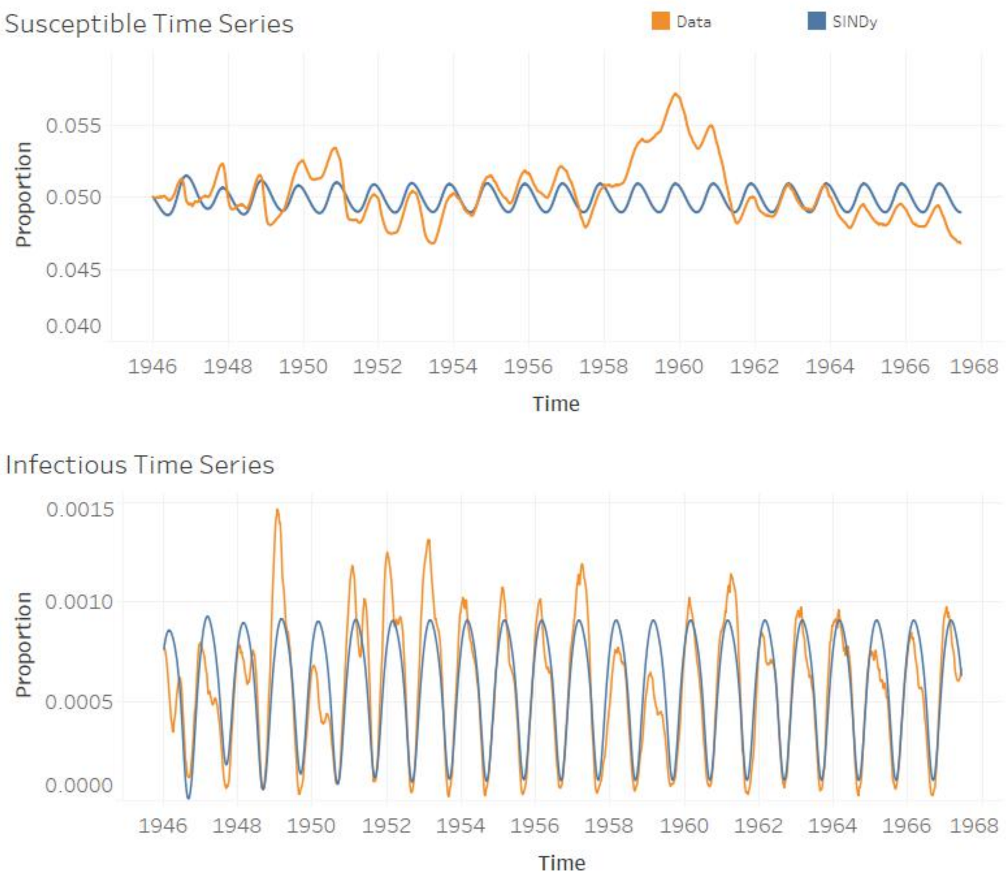

Model Coefficients

Term	S Equation	I Equation
1	-0.147	0.022
S	9.295	-1.247
I	23.803	-8.923
SS	-155.720	23.386
SI	-968.498	377.996
II	2712.909	345.389
SSS	972.259	-146.094
SSI	9664.872	-3616.596
SII	-48146.974	-6361.606
III	-160474.497	-16344.801
B*1	0.005	0.036
B*S	-0.041	-2.204
B*I	0.538	1.940
B*SS	-4.108	44.490
B*SI	12.825	-82.836
B*II	-2629.091	1860.829
B*SSS	58.074	-297.660
B*SSI	-398.769	861.189
B*SII	43755.271	-30140.671
B*III	234042.774	-243853.009

Figure 23: Resulting time series and coefficients from SINDy-discovered models of the chickenpox dataset (with a 3rd order polynomial library) using a relatively low threshold ($\lambda = 0.0001$). The resulting model exhibits a good fit and accurate recovery of attractor class, but all possible terms are active, which is an indicator of an overfit model.

4.4.6 Chickenpox, high sparsity threshold

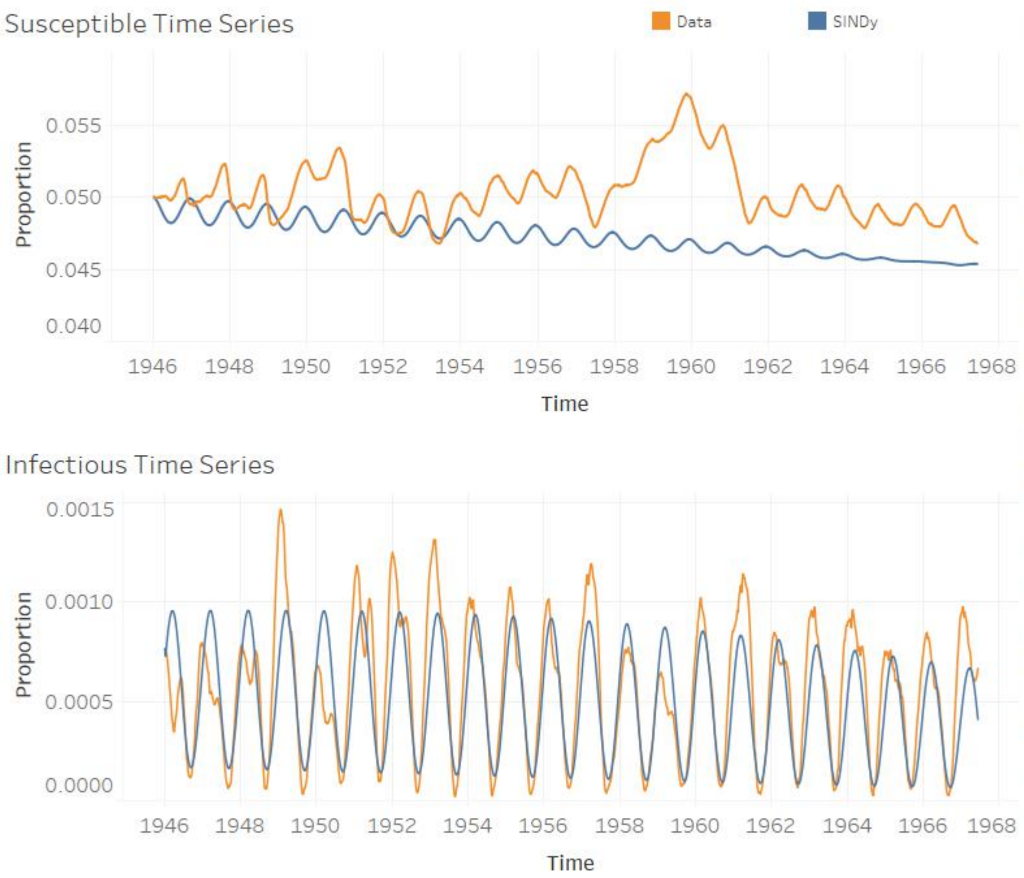

Model Coefficients

Term	S Equation	I Equation
1	0.000	-0.12
S	1.000	7.49
I	0.000	0.00
SS	0.000	-144.44
SI	0.000	0.00
II	0.000	0.00
SSS	0.000	925.93
SSI	0.000	0.00
SII	0.000	0.00
III	0.000	0.00
B*1	0.069	-0.17
B*S	-4.011	10.44
B*I	0.000	0.00
B*SS	77.320	-206.77
B*SI	0.000	0.00
B*II	0.000	0.00
B*SSS	-496.397	1361.04
B*SSI	0.000	0.00
B*SII	0.000	0.00
B*III	0.000	0.00

Figure 24: Resulting time series and coefficients from SINDy-discovered models of the chickenpox dataset (with a 3rd order polynomial library) using a relatively high threshold ($\lambda = 1$). The resulting model exhibits sparsity and recovers the attractor class, but the asymptotic behaviour appears to diverge from the data.

4.4.7 Rubella, parameter plane

Initial Susceptible Values

Lambdas	0.03	0.03714..	0.04428..	0.05142..	0.05857..	0.06571..	0.07285..	0.08	0.08714..	0.09428..	0.101429	0.108571	0.115714	0.122857	0.13
0.0001	-3,582	-3,844	-3,920	-3,959	-3,984	-3,991	-4,027	-4,024	-3,899	-3,669	-3,949		-3,719	-3,728	-3,796
0.000193070	-3,582	-3,844	-3,920	-3,959	-3,984	-3,991	-4,031	-4,024	-3,899	-3,669	-3,949		-3,719	-3,728	-3,796
0.000372759	-3,609	-3,844	-3,920	-3,959	-3,984	-3,991	-4,127	-4,024	-3,880	-3,669	-3,949		-3,719	-3,728	-3,796
0.000719686	-3,557	-3,866	-3,920	-3,959	-3,984	-3,823	-4,127	-4,024	-4,109	-3,669	-3,953		-3,719	-3,728	-3,796
0.001389495	-3,557	-3,866	-3,920	-3,959	-3,924	-3,979	-4,127	-4,080	-3,526	-3,669	-3,953		-3,740	-3,716	-4,085
0.002682696	-3,820	-3,592	-4,191	-3,959	-3,924	-3,998	-3,827	-3,944	-3,933	-3,916	-3,858	-3,819	-3,780	-3,827	-4,143
0.005179475	-3,498	-3,673	-3,906	-3,959	-3,837	-4,025	-4,291	-4,176	-4,023	-3,911	-3,855	-3,785	-3,934	-3,960	-3,830
0.01	-3,605	-3,586	-3,661	-3,706	-3,949	-3,717	-4,043	-4,139	-3,937	-3,902	-3,852	-3,785	-3,726	-3,960	-4,032
0.019306977	-3,419	-3,586	-3,653	-3,626	-3,762	-3,810	-3,709	-3,945	-3,991	-3,884	-3,877	-3,922	-3,734	-3,658	-3,767
0.037275937	-3,431	-3,474	-3,576	-3,647	-3,696	-3,708	-3,799	-3,852	-3,743	-4,021	-3,823	-4,005	-4,002	-3,658	-3,575
0.071968567	-3,884	-4,092	-3,435	-3,762	-3,782	-3,799	-3,810	-3,806	-3,724	-4,035	-3,941	-3,801	-4,107	-3,721	-3,609
0.138949549	-4,107	-4,090	-4,093	-4,096	-3,754	-3,786	-3,802	-3,820	-4,120	-4,129	-4,038	-3,887	-3,847	-3,717	-3,594
0.268269580	-4,107	-3,706	-4,091	-4,094	-4,099	-4,105	-3,932	-3,848	-4,129	-4,154	-4,112	-3,887	-3,769	-3,715	-3,611
0.517947468	-2,555	-2,437	-4,093	-3,647	-3,834	-3,868	-3,928	-4,144	-4,149	-4,089	-4,129	-4,136	-3,857	-3,693	-3,711
1	-2,556	-2,437	-4,093	-4,093	-4,096	-4,098	-4,107	-4,116	-4,120	-4,089	-4,017	-3,908	-3,788	-3,815	-3,841

Figure 25: The AIC values for SINDy models across a range of both initial susceptible and threshold values, utilizing the rubella dataset and a 3rd order polynomial library. Darker colour refers to a lower AIC value. Cells without value refer to a model which, when simulated, resulted in a diverging time series.

4.4.8 Rubella, low sparsity threshold

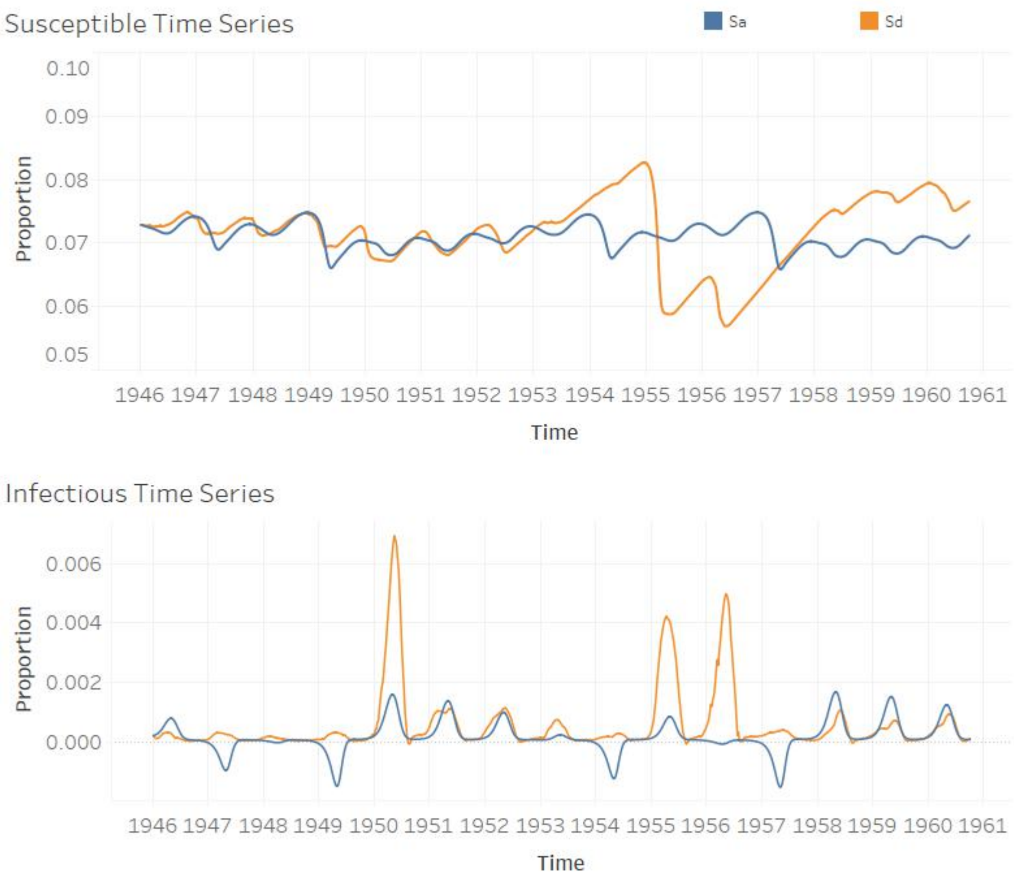

Model Coefficients

Term	S Equation	I Equation
1	0.028	-0.032
S	-0.062	1.342
I	-6.200	4.909
SS	13.168	-18.896
SI	196.458	-123.940
II	-95.626	-13.320
SSS	-53.433	88.193
SSI	-1553.162	948.094
SII	466.684	380.859
III	9692.752	404.334
B*1	0.040	-0.026
B*S	-1.564	1.120
B*I	-1.924	-0.871
B*SS	20.331	-15.842
B*SI	76.350	48.285
B*II	-379.235	-189.057
B*SSS	-87.011	74.477
B*SSI	-603.043	-452.256
B*SII	28.623	1826.271
B*III	48943.382	8731.623

Figure 26: Resulting time series and coefficients from SINDy-discovered models of the rubella dataset (with a 3rd order polynomial library) using a relatively low threshold ($\lambda = 0.0001$). The resulting model attempts to capture the peaks of the underlying multi-annual attractor, but at the cost of a high number of active nonlinearities. Note that the negative peaks in the infectious time series are not biologically relevant, but can be adjusted using a constraint when simulating.

4.4.9 Rubella, high sparsity threshold

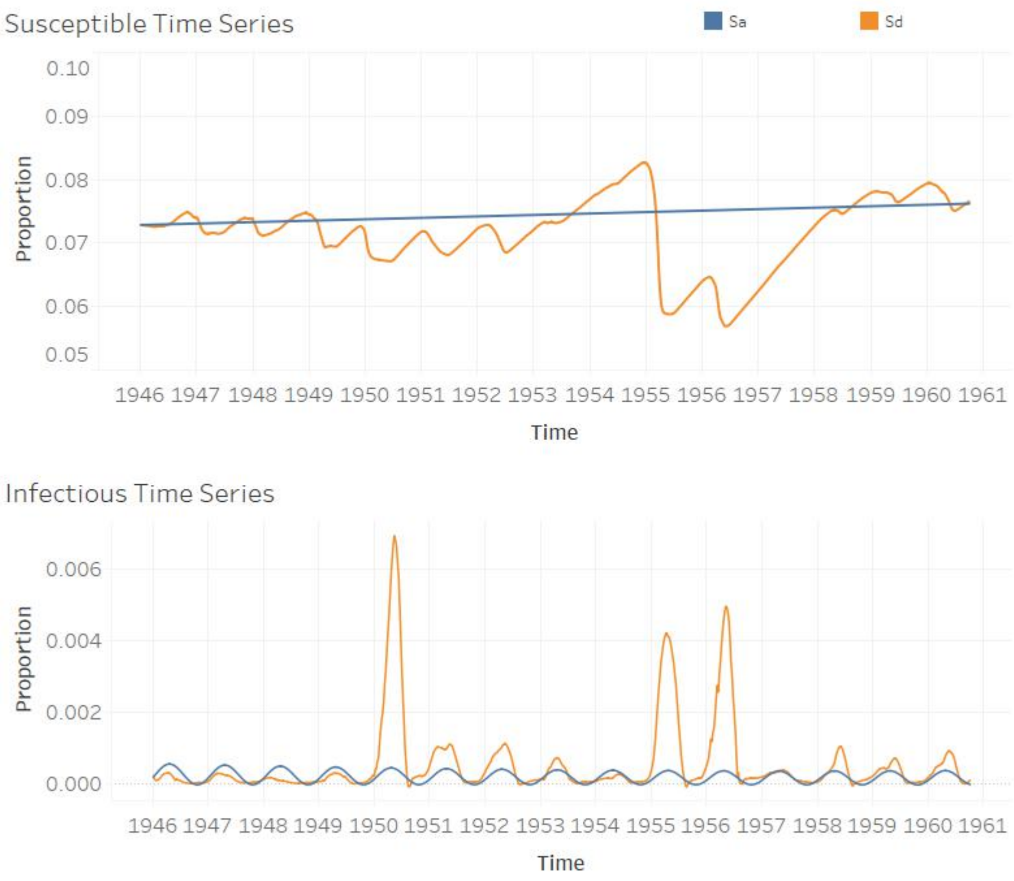

Model Coefficients

Term	S Equation	I Equation
1	0.000	-0.181
S	1.000	8.334
I	0.000	0.000
SS	0.000	-125.386
SI	0.000	0.000
II	0.000	0.000
SSS	0.000	619.668
SSI	0.000	0.000
SII	0.000	0.000
III	0.000	0.000
B*1	0.000	-0.122
B*S	0.000	6.080
B*I	0.000	0.000
B*SS	0.000	-96.754
B*SI	0.000	0.000
B*II	0.000	0.000
B*SSS	0.000	498.833
B*SSI	0.000	0.000
B*SII	0.000	0.000
B*III	0.000	0.000

Figure 27: Resulting time series and coefficients from SINDy-discovered models of the rubella dataset (with a 3rd order polynomial library) using a relatively high threshold ($\lambda = 1$). The resulting model exhibits sparsity but is linear in the susceptible time series and has an annual oscillation in the infectious time series, neither of which match the dynamics of the system.

5 Out-of-sample prediction

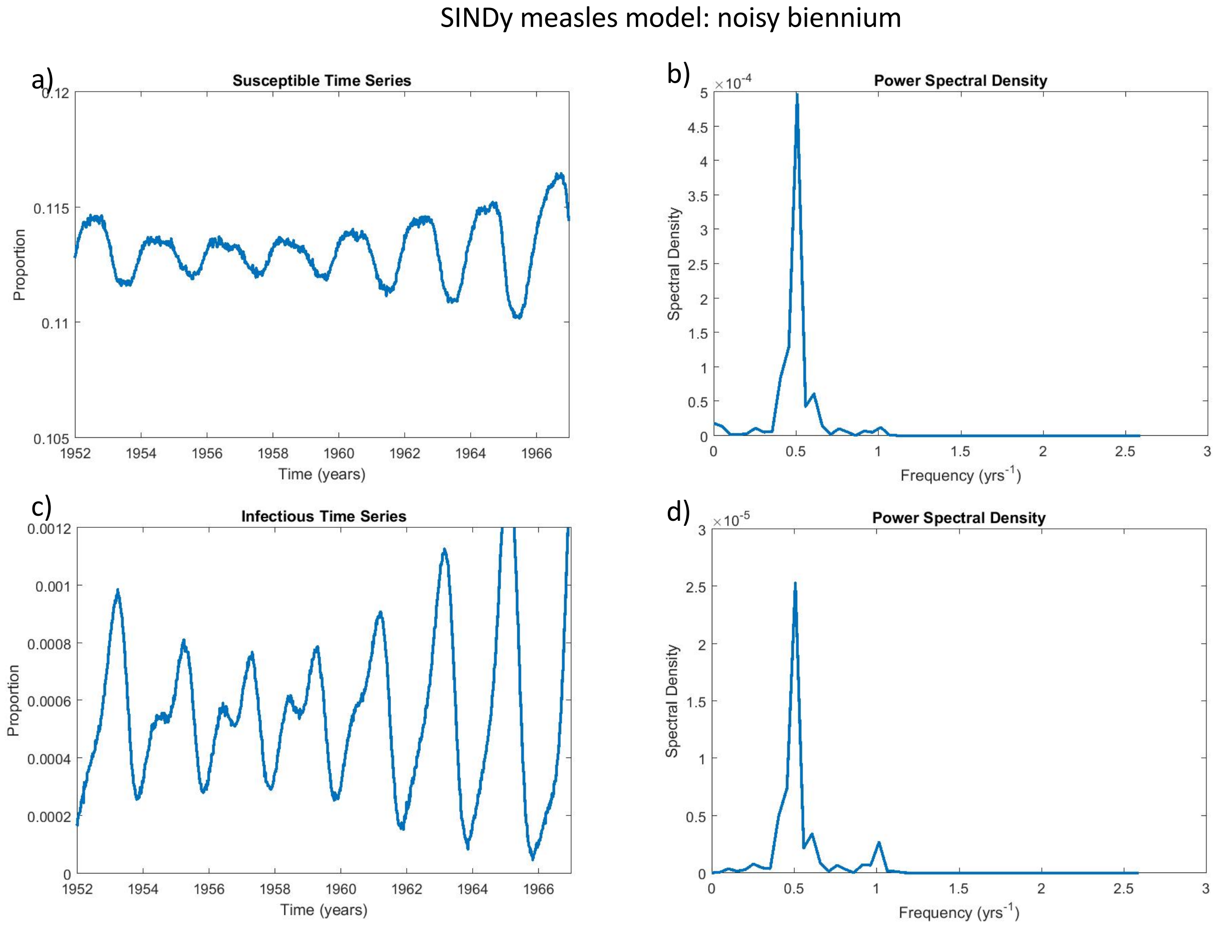

Figure 28: The SINDy measles biennium is robust to the addition of noise. This figure depicts the simulated timeseries of the SINDy measles model under additive noise for a different random number seed than Figure 8 of the main text or for other Supplementary Figures showing similar results: subpanels show the proportion of susceptible (a) and infected (c) individuals over time and the corresponding power spectral density plots for the susceptible (b) and infectious (d) time series. The power spectral density plots show strong power at a frequency of 0.5/year and a lesser peak at 1/year, corresponding to a prominent biennial cycle. White noise with a coefficient of 1.5×10^{-3} was added to the right-hand side of the SINDy-discovered system of differential equations to generate these plots. See Methods for details about computation of the power spectral density.

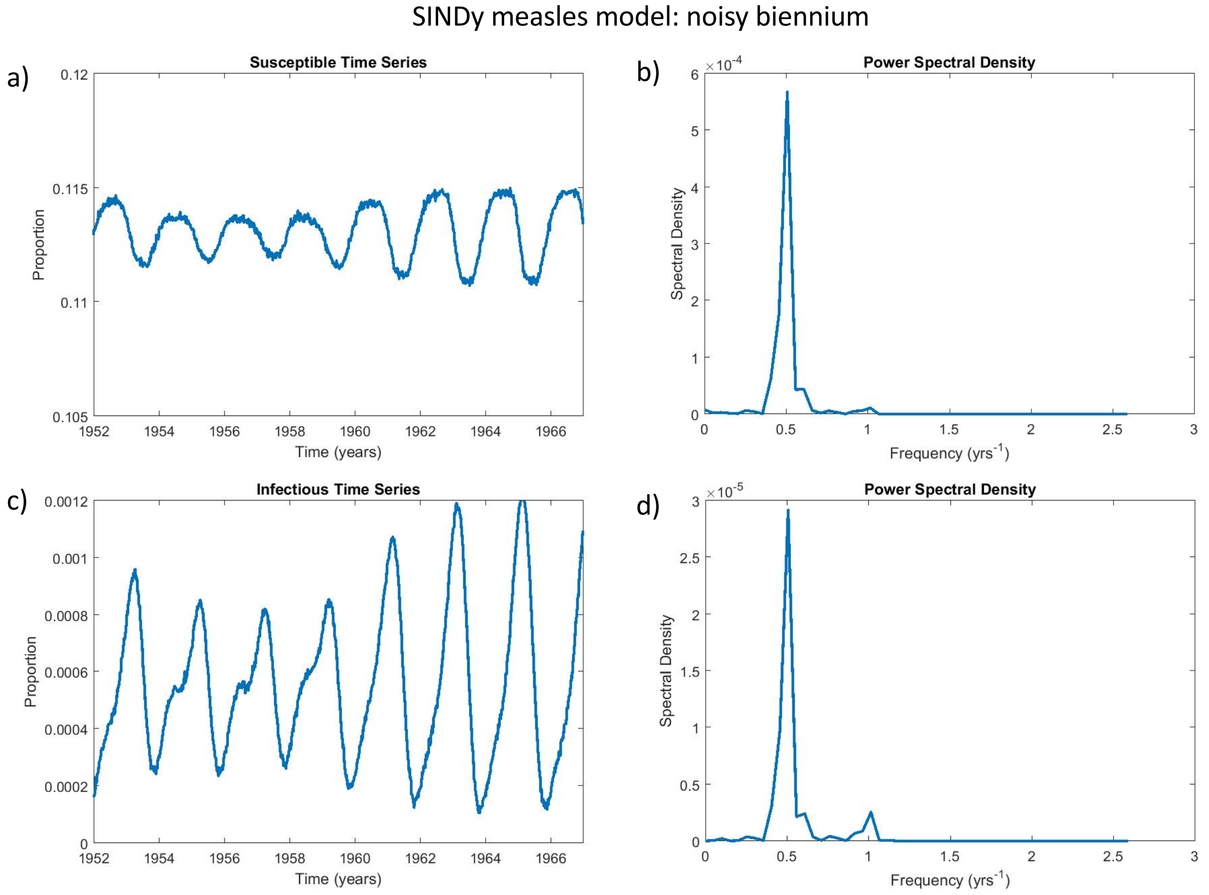

Figure 29: The SINDy measles biennium is robust to the addition of noise. This figure depicts the simulated timeseries of the SINDy measles model under additive noise for a different random number seed than Figure 8 of the main text or for other Supplementary Figures showing similar results: subpanels show the proportion of susceptible (a) and infected (c) individuals over time and the corresponding power spectral density plots for the susceptible (b) and infectious (d) time series. The power spectral density plots show strong power at a frequency of 0.5/year and a lesser peak at 1/year, corresponding to a prominent biennial cycle. White noise with a coefficient of 1.5×10^{-3} was added to the right-hand side of the SINDy-discovered system of differential equations to generate these plots. See Methods for details about computation of the power spectral density.

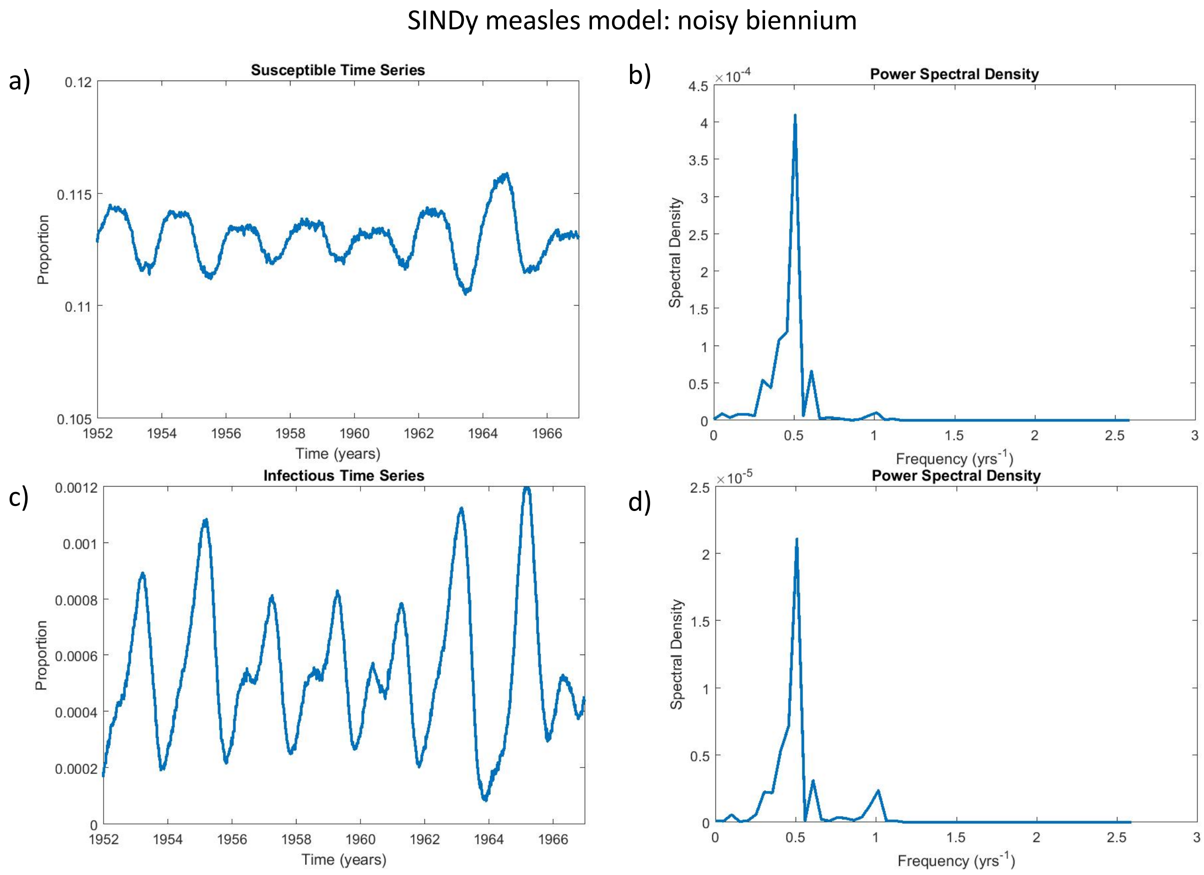

Figure 30: The SINDy measles biennium is robust to the addition of noise. This figure depicts the simulated timeseries of the SINDy measles model under additive noise for a different random number seed than Figure 8 of the main text or for other Supplementary Figures showing similar results: subpanels show the proportion of susceptible (a) and infected (c) individuals over time and the corresponding power spectral density plots for the susceptible (b) and infectious (d) time series. The power spectral density plots show strong power at a frequency of 0.5/year and a lesser peak at 1/year, corresponding to a prominent biennial cycle. White noise with a coefficient of 1.5×10^{-3} was added to the right-hand side of the SINDy-discovered system of differential equations to generate these plots. See Methods for details about computation of the power spectral density.

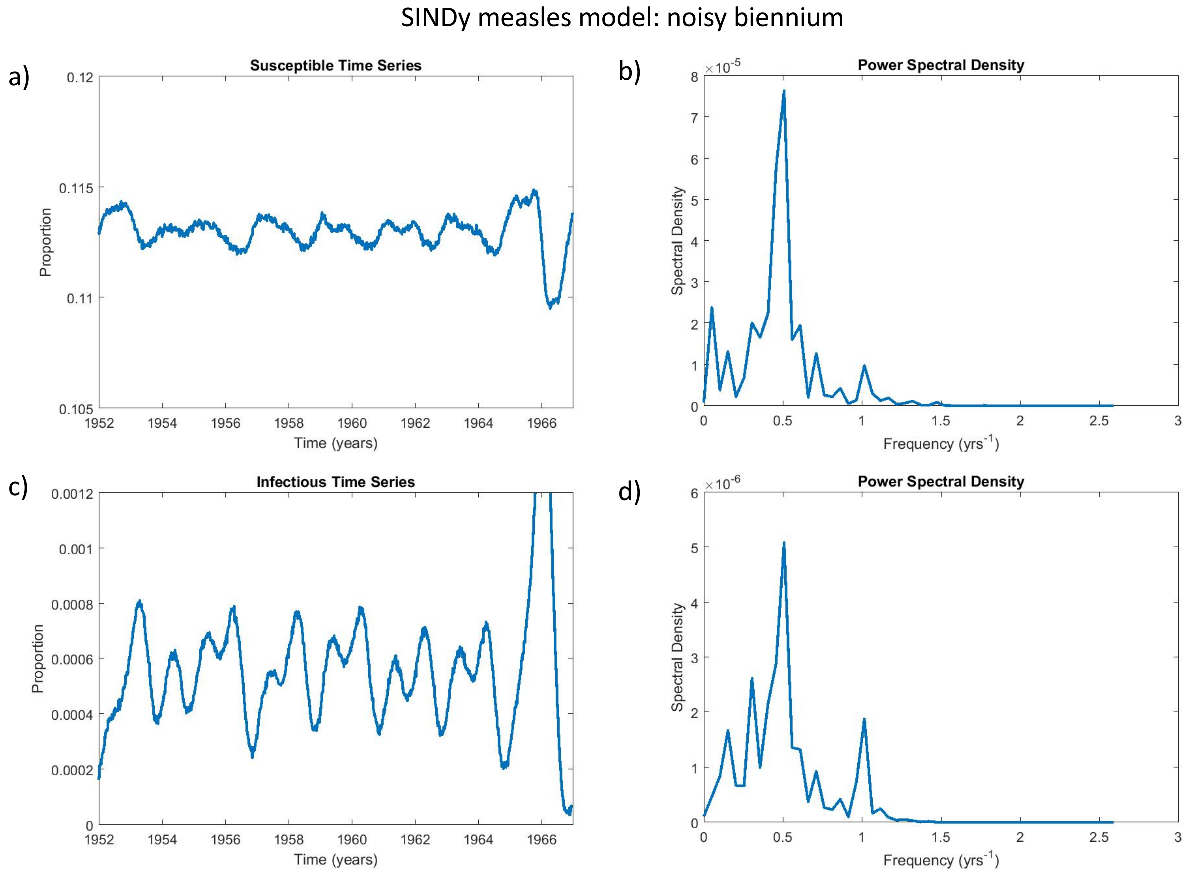

Figure 31: The SINDy measles biennium is robust to the addition of noise. This figure depicts the simulated timeseries of the SINDy measles model under additive noise for a different random number seed than Figure 8 of the main text or for other Supplementary Figures showing similar results: subpanels show the proportion of susceptible (a) and infected (c) individuals over time and the corresponding power spectral density plots for the susceptible (b) and infectious (d) time series. The power spectral density plots show strong power at a frequency of 0.5/year and a lesser peak at 1/year, corresponding to a prominent biennial cycle. White noise with a coefficient of 1.5×10^{-3} was added to the right-hand side of the SINDy-discovered system of differential equations to generate these plots. See Methods for details about computation of the power spectral density.

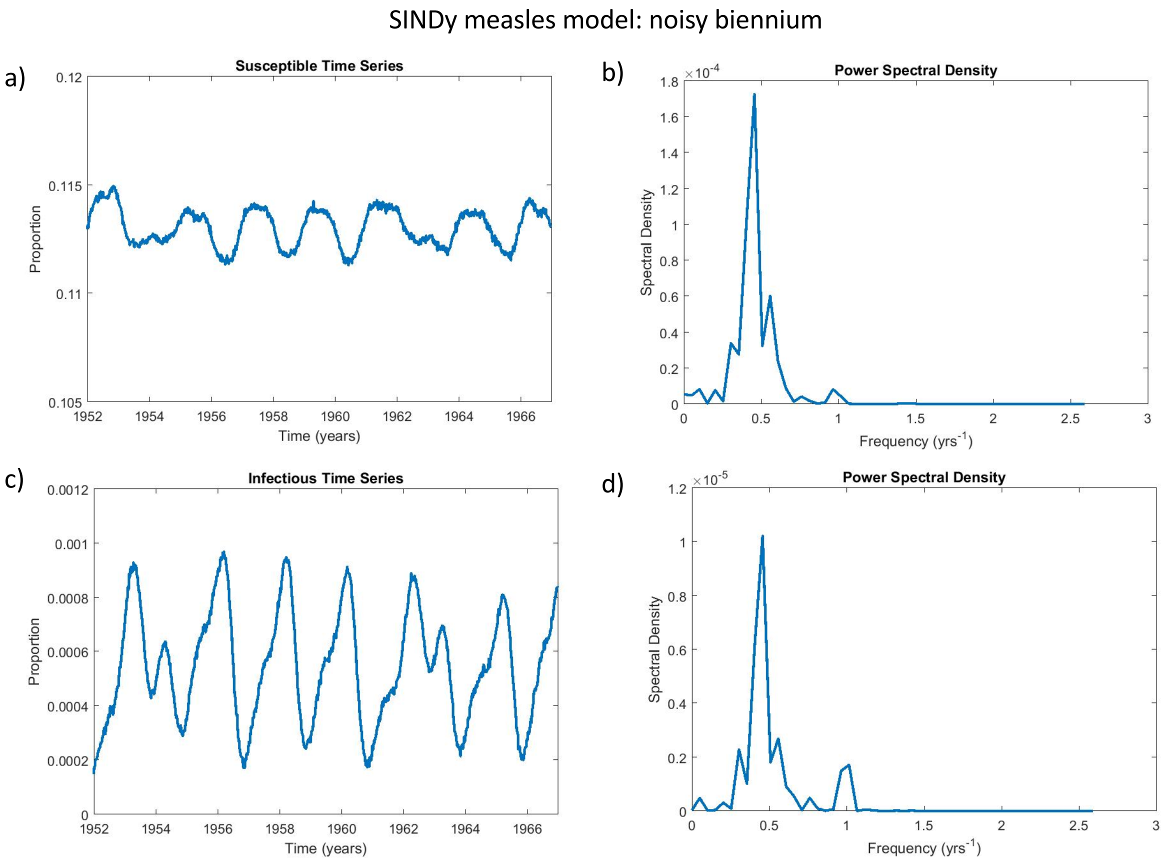

Figure 32: The SINDy measles biennium is robust to the addition of noise. This figure depicts the simulated timeseries of the SINDy measles model under additive noise for a different random number seed than Figure 8 of the main text or for other Supplementary Figures showing similar results: subpanels show the proportion of susceptible (a) and infected (c) individuals over time and the corresponding power spectral density plots for the susceptible (b) and infectious (d) time series. The power spectral density plots show strong power at a frequency of 0.5/year and a lesser peak at 1/year, corresponding to a prominent biennial cycle. White noise with a coefficient of 1.5×10^{-3} was added to the right-hand side of the SINDy-discovered system of differential equations to generate these plots. See Methods for details about computation of the power spectral density.

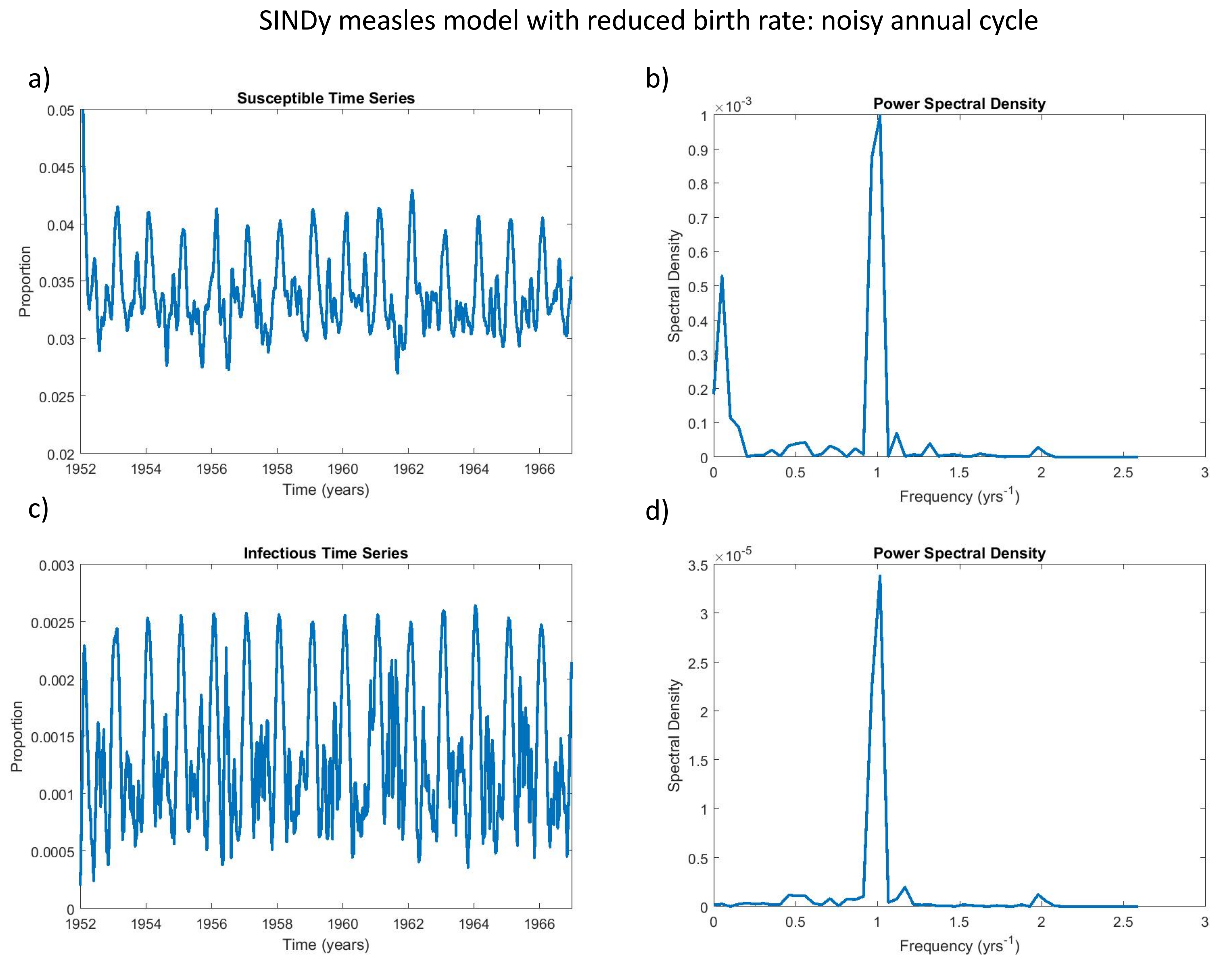

Figure 33: The SINDy measles model predicts a noisy annual attractor when the recruitment rate is reduced. This figure depicts the simulated timeseries of the SINDy measles model with a reduced birth rate, under additive noise for a different random number seed than Figure 9 of the main text or for other Supplementary Figures showing similar results: subpanels show the proportion of susceptible (a) and infected (c) individuals over time and the corresponding power spectral density plots for the susceptible (b) and infectious (d) time series. The power spectral density plots show strong power at a frequency of 0.5/year and a lesser peak at 1/year, corresponding to a prominent biennial cycle. White noise with a coefficient of 1.5×10^{-3} was added to the right-hand side of the SINDy-discovered system of differential equations to generate these plots. See Methods for details about computation of the power spectral density. To simulate a reduced recruitment rate of newly-born susceptible individuals from 2.60/year to 1.36/year (expressed as the total fertility rate of susceptible offspring) between 1948-1967 and 1968-1988 in the United Kingdom due to falling birth rates and mass vaccination the coefficient of S was changed from 0.606 to 0.317.

SINDy measles model with reduced birth rate: noisy annual cycle

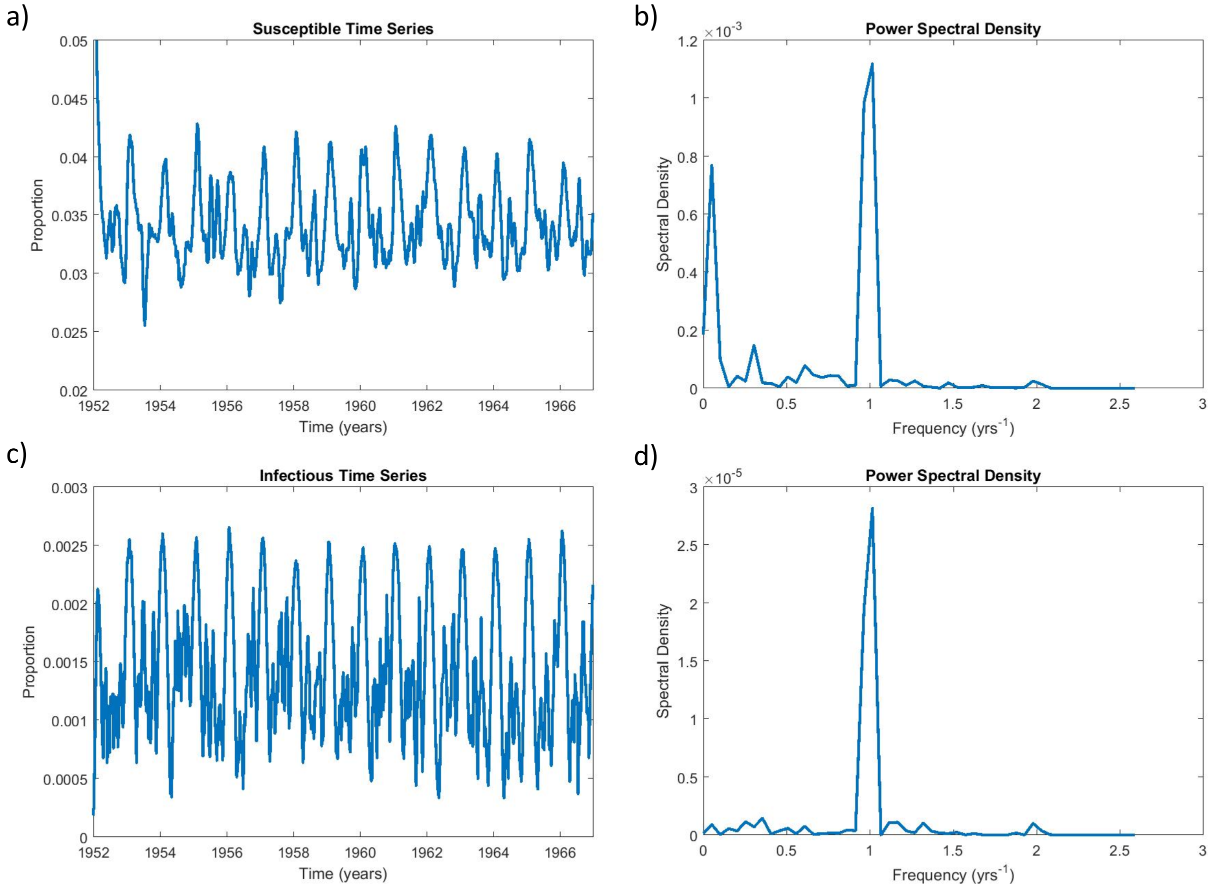

Figure 34: The SINDy measles model predicts a noisy annual attractor when the recruitment rate is reduced. This figure depicts the simulated timeseries of the SINDy measles model with a reduced birth rate, under additive noise for a different random number seed than Figure 9 of the main text or for other Supplementary Figures showing similar results: subpanels show the proportion of susceptible (a) and infected (c) individuals over time and the corresponding power spectral density plots for the susceptible (b) and infectious (d) time series. The power spectral density plots show strong power at a frequency of 0.5/year and a lesser peak at 1/year, corresponding to a prominent biennial cycle. White noise with a coefficient of 1.5×10^{-3} was added to the right-hand side of the SINDy-discovered system of differential equations to generate these plots. See Methods for details about computation of the power spectral density. To simulate a reduced recruitment rate of newly-born susceptible individuals from 2.60/year to 1.36/year (expressed as the total fertility rate of susceptible offspring) between 1948-1967 and 1968-1988 in the United Kingdom due to falling birth rates and mass vaccination the coefficient of S was changed from 0.606 to 0.317.

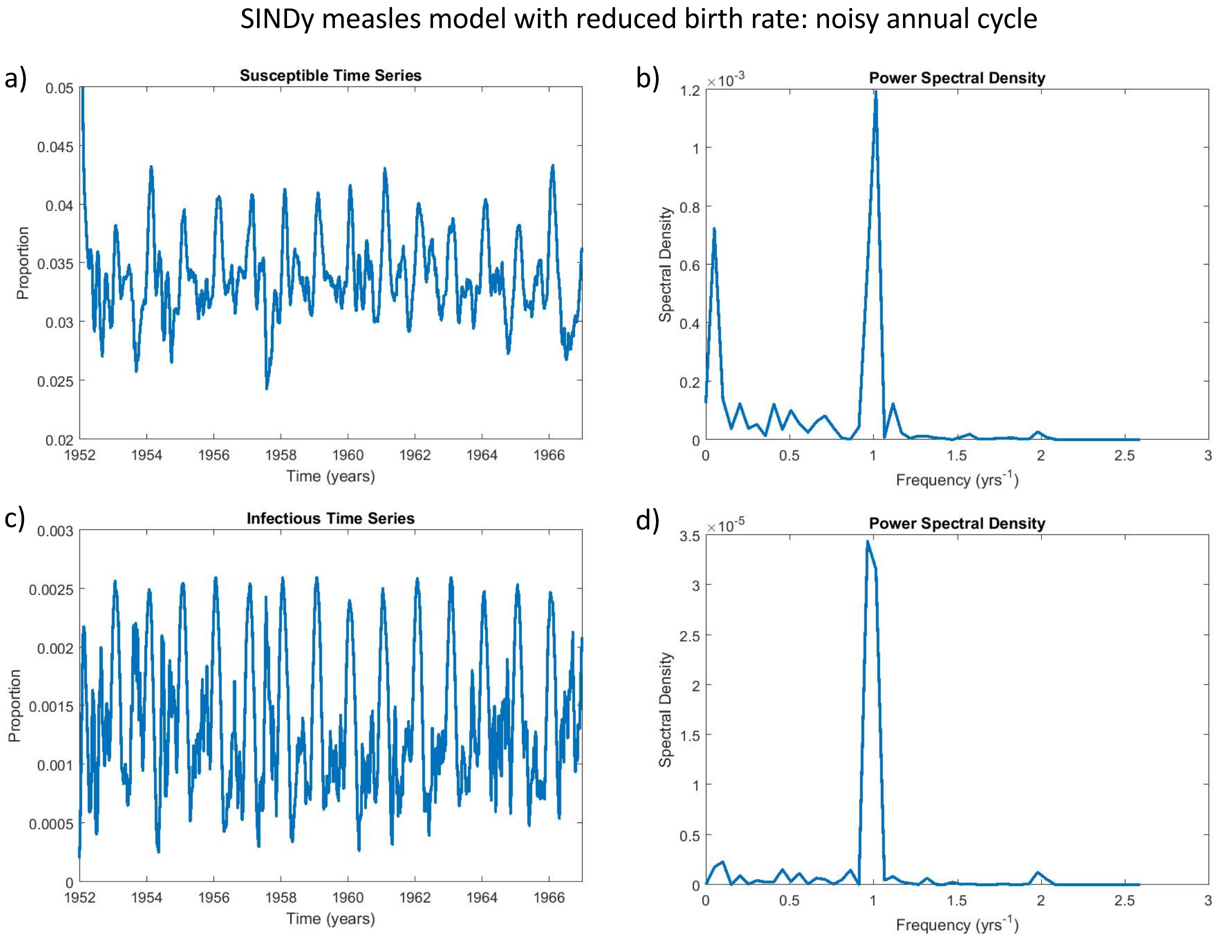

Figure 35: The SINDy measles model predicts a noisy annual attractor when the recruitment rate is reduced. This figure depicts the simulated timeseries of the SINDy measles model with a reduced birth rate, under additive noise for a different random number seed than Figure 9 of the main text or for other Supplementary Figures showing similar results: subpanels show the proportion of susceptible (a) and infected (c) individuals over time and the corresponding power spectral density plots for the susceptible (b) and infectious (d) time series. The power spectral density plots show strong power at a frequency of 0.5/year and a lesser peak at 1/year, corresponding to a prominent biennial cycle. White noise with a coefficient of 1.5×10^{-3} was added to the right-hand side of the SINDy-discovered system of differential equations to generate these plots. See Methods for details about computation of the power spectral density. To simulate a reduced recruitment rate of newly-born susceptible individuals from 2.60/year to 1.36/year (expressed as the total fertility rate of susceptible offspring) between 1948-1967 and 1968-1988 in the United Kingdom due to falling birth rates and mass vaccination the coefficient of S was changed from 0.606 to 0.317.

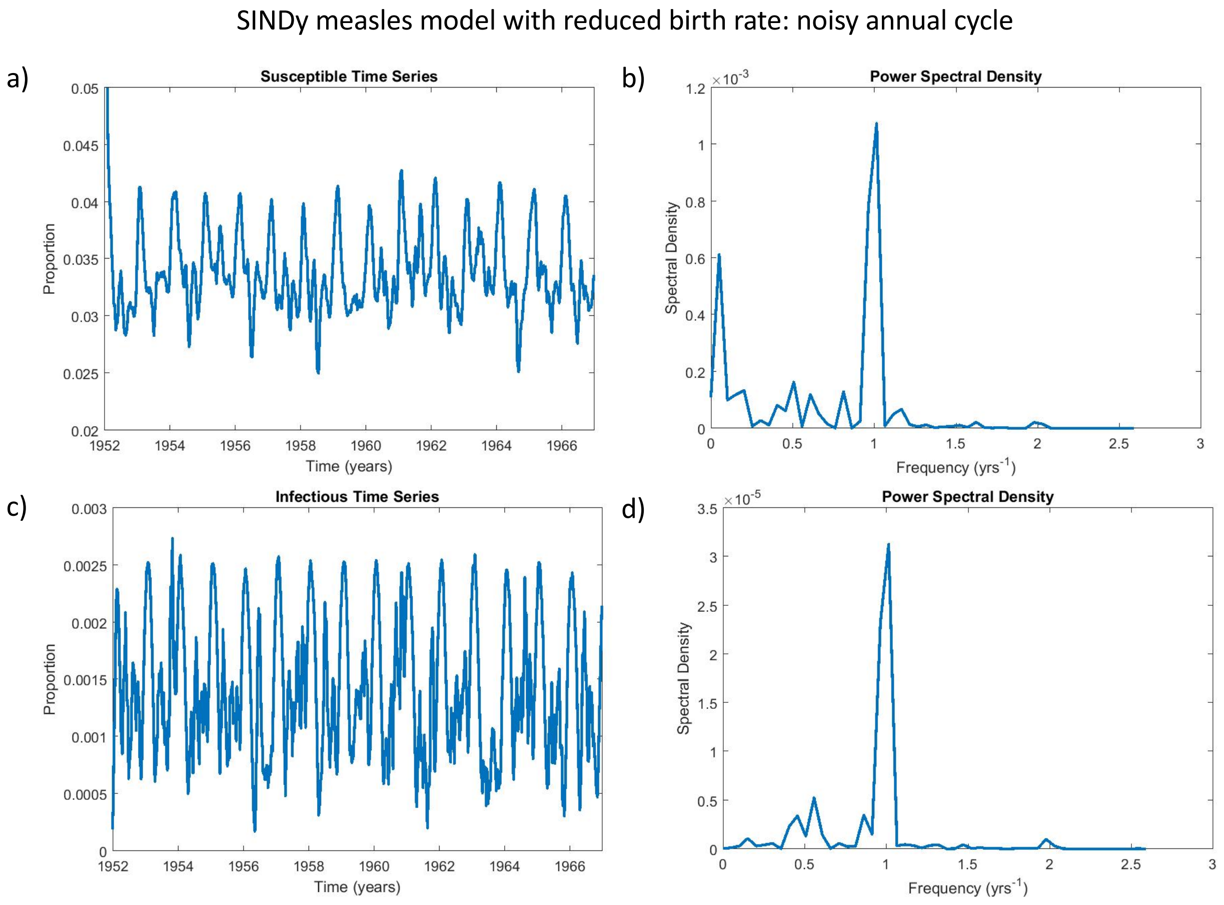

Figure 36: The SINDy measles model predicts a noisy annual attractor when the recruitment rate is reduced. This figure depicts the simulated timeseries of the SINDy measles model with a reduced birth rate, under additive noise for a different random number seed than Figure 9 of the main text or for other Supplementary Figures showing similar results: subpanels show the proportion of susceptible (a) and infected (c) individuals over time and the corresponding power spectral density plots for the susceptible (b) and infectious (d) time series. The power spectral density plots show strong power at a frequency of 0.5/year and a lesser peak at 1/year, corresponding to a prominent biennial cycle. White noise with a coefficient of 1.5×10^{-3} was added to the right-hand side of the SINDy-discovered system of differential equations to generate these plots. See Methods for details about computation of the power spectral density. To simulate a reduced recruitment rate of newly-born susceptible individuals from 2.60/year to 1.36/year (expressed as the total fertility rate of susceptible offspring) between 1948-1967 and 1968-1988 in the United Kingdom due to falling birth rates and mass vaccination the coefficient of S was changed from 0.606 to 0.317.

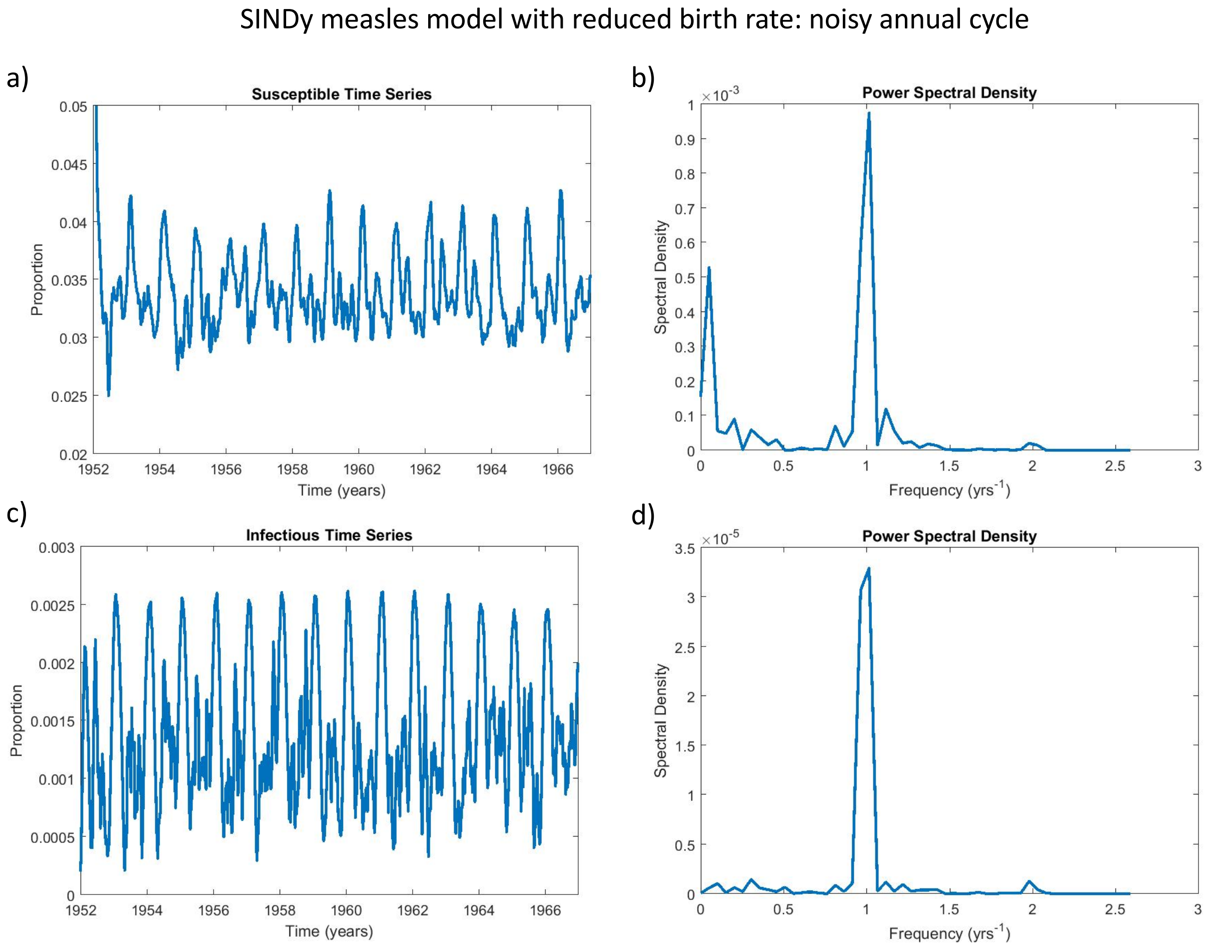

Figure 37: The SINDy measles model predicts a noisy annual attractor when the recruitment rate is reduced. This figure depicts the simulated timeseries of the SINDy measles model with a reduced birth rate, under additive noise for a different random number seed than Figure 9 of the main text or for other Supplementary Figures showing similar results: subpanels show the proportion of susceptible (a) and infected (c) individuals over time and the corresponding power spectral density plots for the susceptible (b) and infectious (d) time series. The power spectral density plots show strong power at a frequency of 0.5/year and a lesser peak at 1/year, corresponding to a prominent biennial cycle. White noise with a coefficient of 1.5×10^{-3} was added to the right-hand side of the SINDy-discovered system of differential equations to generate these plots. See Methods for details about computation of the power spectral density. To simulate a reduced recruitment rate of newly-born susceptible individuals from 2.60/year to 1.36/year (expressed as the total fertility rate of susceptible offspring) between 1948-1967 and 1968-1988 in the United Kingdom due to falling birth rates and mass vaccination the coefficient of S was changed from 0.606 to 0.317.

6 Birth rate data

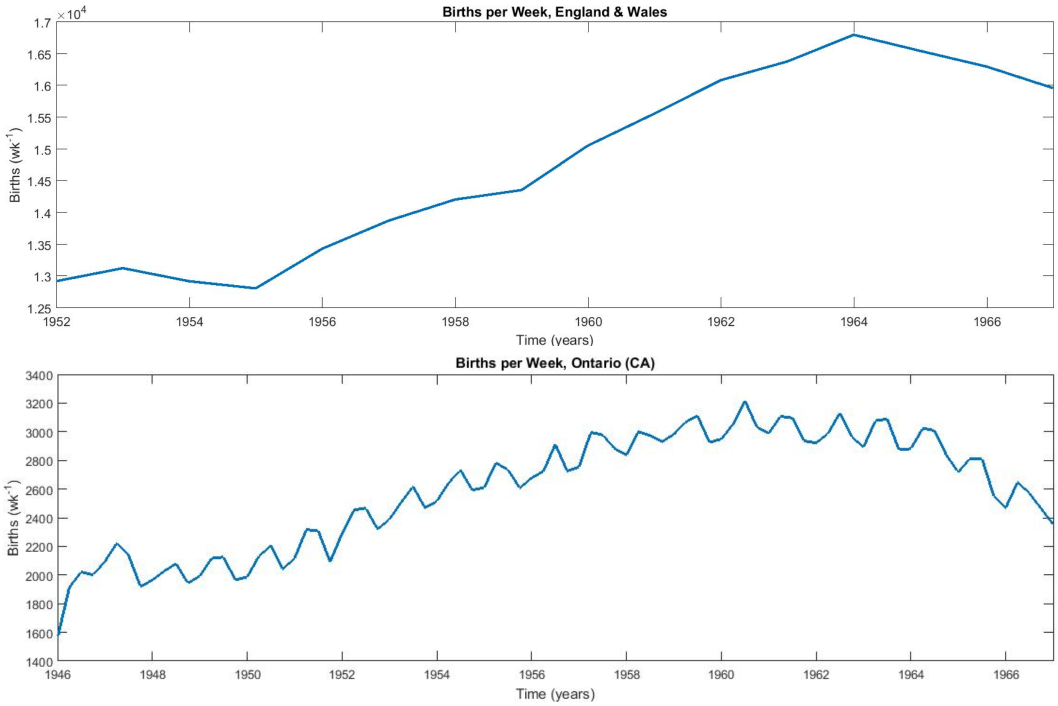

Figure 38: Birth rate data used for susceptible reconstruction, for the UK (top) and Ontario (bottom).

7 Susceptible Reconstruction

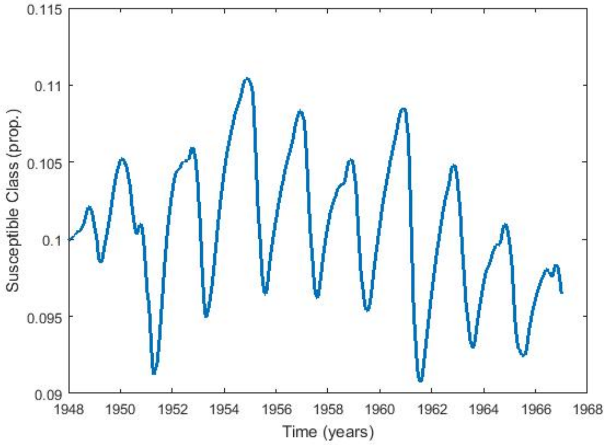

(a) Measles (UK)

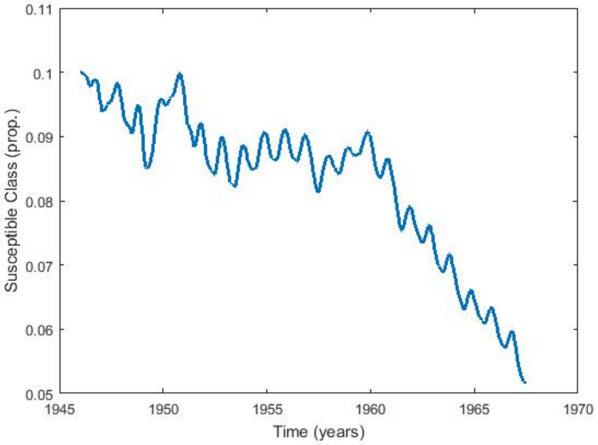

(b) chickenpox (Ontario)

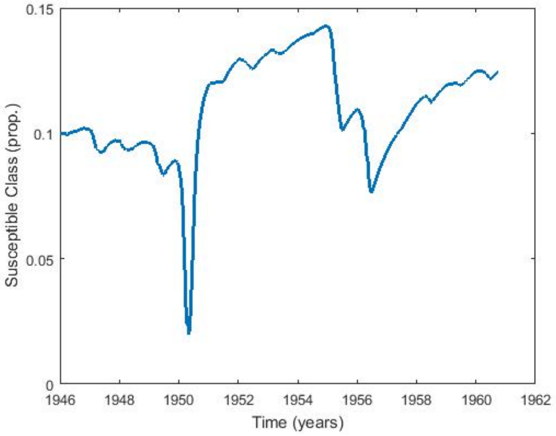

(c) Rubella (Ontario)

Figure 39: Suceptible reconstructions for measles (a), chickenpox (b), and rubella (c) using the global regression method.

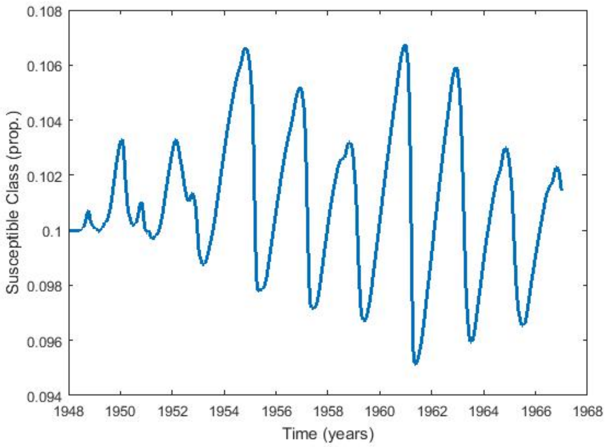

(a) Measles (UK)

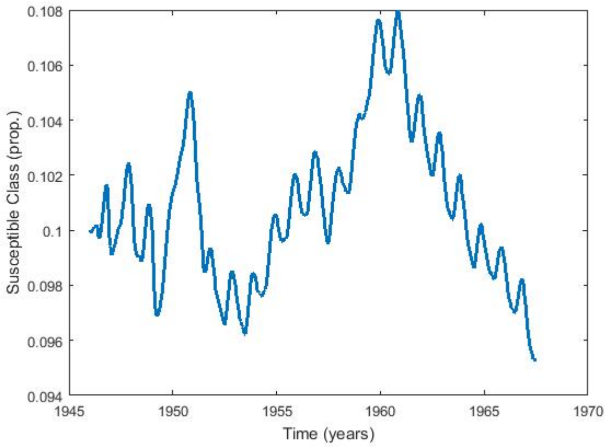

(b) chickenpox (Ontario)

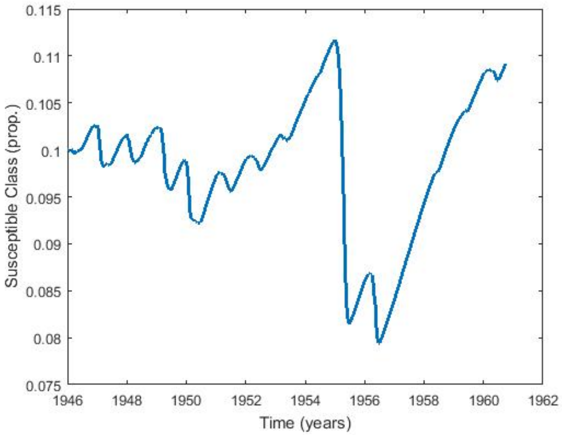

(c) Rubella (Ontario)

Figure 40: Suceptible reconstructions for measles (a), chickenpox (b), and rubella (c) using the locally linear regression method.

8 Transmission rate reconstruction

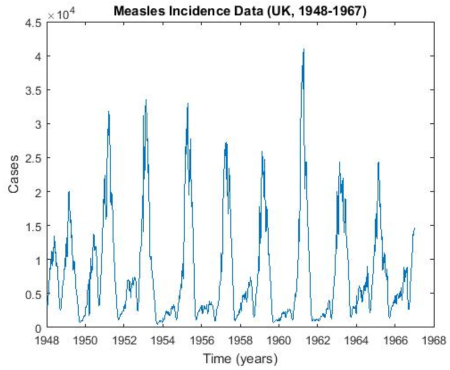

(a) Incidence (measles)

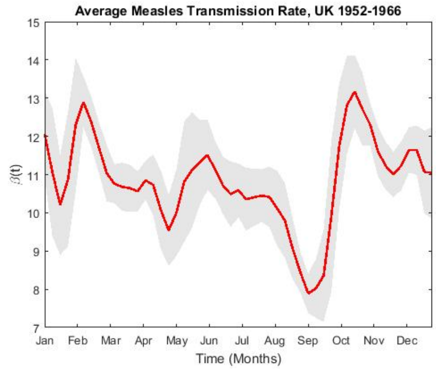

(b) Transmission rate estimate (measles)

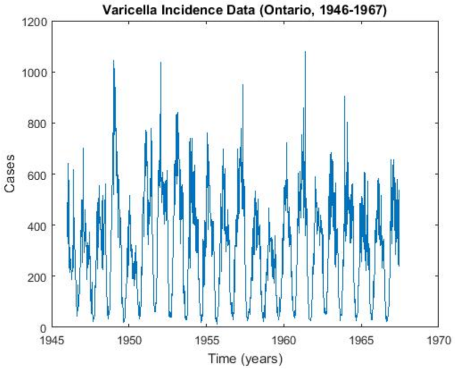

(c) Incidence (chickenpox)

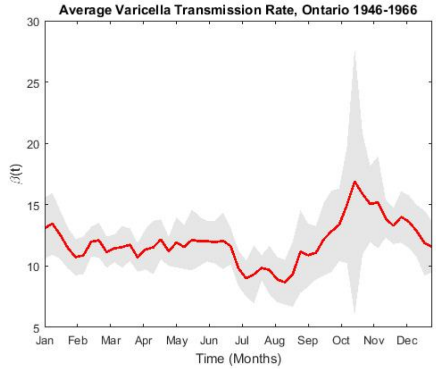

(d) Transmission rate estimate (chickenpox)

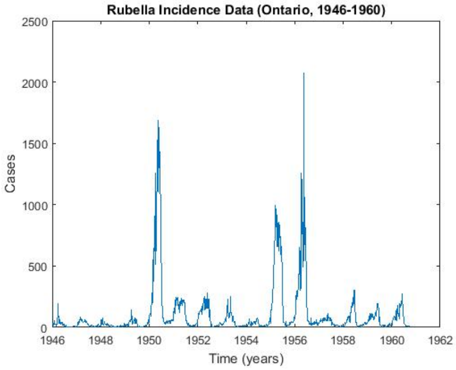

(e) Incidence (rubella)

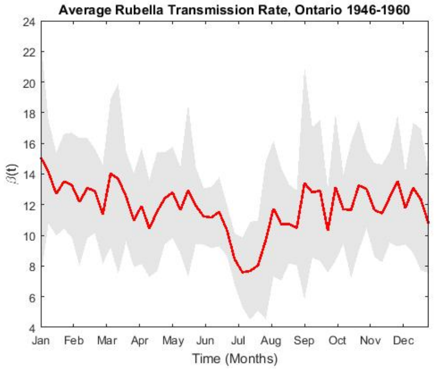

(f) Transmission rate estimate (rubella)

Figure 41: Reconstructed time-varying transmission rate $\beta(t)$ for three infectious diseases. Subpanels show weekly case notifications for (a) measles, (c) chickenpox, and (e) rubella and (b, d, f) their corresponding reconstructed $\beta(t)$. Red line in (b,d,f) shows mean value of reconstruction, and shaded areas show +/- one standard deviation.

9 SIR Model Fitting

**Symbol**	$\mathcal{R}_0$	γ	β_1	μ	ϕ
**Description**	Basic Rep. Number	Recovery rate	Forcing amplitude	Birth/death rate	Forcing phase
**Range**	6 - 16	0.55 - 1.25	0.05 - 0.35	3×10^{-4} - 6×10^{-4}	0-51.5
**Step Size**	0.5	0.05	0.025	5×10^{-5}	0.5

Table 1: Parameters, ranges, and step sizes used for fitting discrete SIR model (Eqs. 1-3 of main text) to empirical data for each of the three disease datasets.

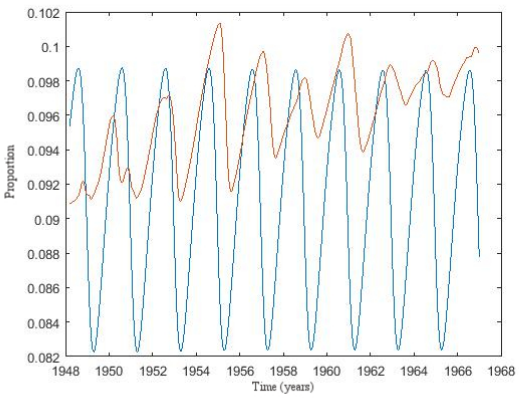

(a) Susceptibles

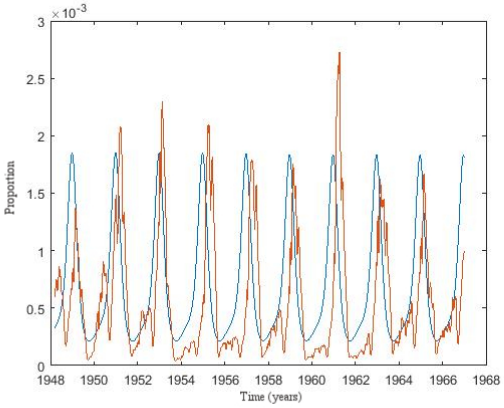

(b) Infectious

β_0	β_1	γ	μ
7.7	0.03	0.7	0.0005

.

Figure 42: Comparison between the empirical data and a fitted SIR model for the measles dataset. Parameters used when simulating the discrete SIR model (Eqs. 1-3 of main text) were obtained by sweeping across the grid defined by the ranges and step sizes given in Table 1, selecting parameters that gave the model that minimized residual error.

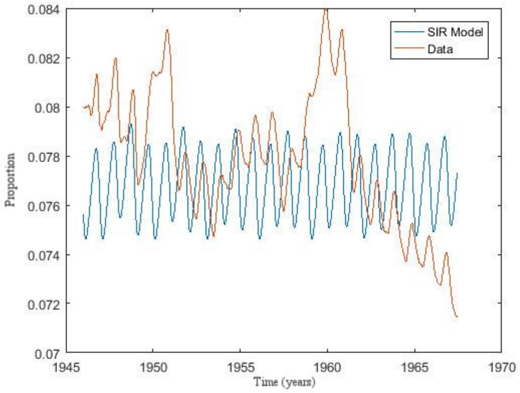

(a) Susceptibles

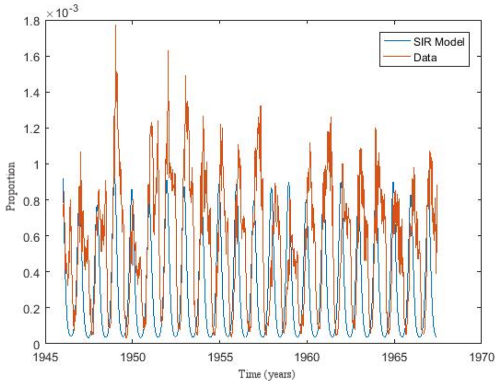

(b) Infectious

β_0	β_1	γ	μ
7.8	0.3	0.6	0.0002

.

Figure 43: Comparison between the empirical data and a fitted SIR model for the chickenpox dataset. Parameters used when simulating the discrete SIR model (Eqs. 1-3 of main text) were obtained by sweeping across the grid defined by the ranges and step sizes given in Table 1, selecting parameters that gave the model that minimized residual error.

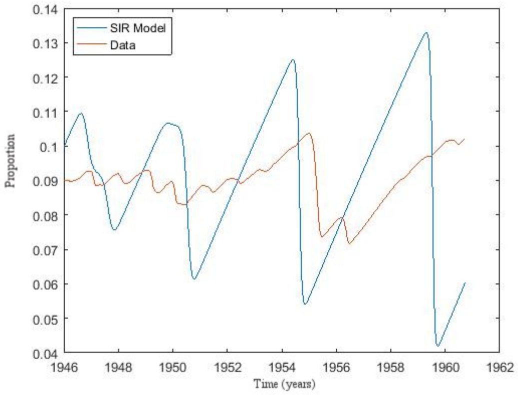

(a) Susceptibles

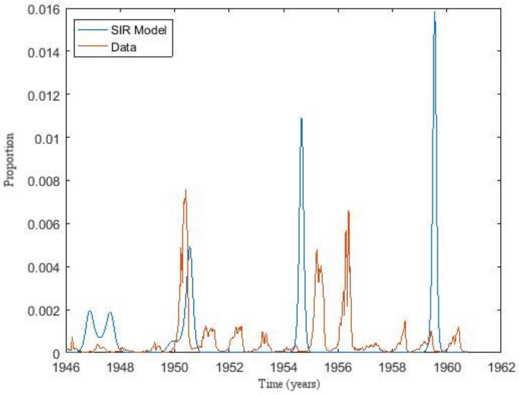

(b) Infectious

β_0	β_1	γ	μ
7.7	0.15	0.7	0.0004

.

Figure 44: Comparison between the empirical data and a fitted SIR model for the rubella dataset. Parameters used when simulating the discrete SIR model (Eqs. 1-3 of main text) were obtained by sweeping across the grid defined by the ranges and step sizes given in Table 1, selecting parameters that gave the model that minimized residual error.
